# Mix and Match: Promoters and Terminators for Tuning Gene Expression in the Methylotrophic Yeast *Ogataea polymorpha*

**DOI:** 10.3389/fbioe.2022.876316

**Published:** 2022-05-10

**Authors:** Katrin Wefelmeier, Birgitta E. Ebert, Lars M. Blank, Simone Schmitz

**Affiliations:** ^1^ IAMB—Institute of Applied Microbiology, ABBt, Aachen Biology and Biotechnology, RWTH Aachen University, Aachen, Germany; ^2^ Australian Institute for Bioengineering and Nanotechnology, The University of Queensland, Brisbane, QLD, Australia

**Keywords:** Ogataea polymorpha, Hansenula polymorpha, methylotrophic yeast, promoters, terminators, genetic tools

## Abstract

The yeast *Ogataea polymorpha* is an upcoming host for bio-manufacturing due to its unique physiological properties, including its broad substrate spectrum, and particularly its ability to utilize methanol as the sole carbon and energy source. However, metabolic engineering tools for *O. polymorpha* are still rare. In this study we characterized the influence of 6 promoters and 15 terminators on gene expression throughout batch cultivations with glucose, glycerol, and methanol as carbon sources as well as mixes of these carbon sources. For this characterization, a short half-life Green Fluorescent Protein (GFP) variant was chosen, which allows a precise temporal resolution of gene expression. Our promoter studies revealed how different promoters do not only influence the expression strength but also the timepoint of maximal expression. For example, the expression strength of the catalase promoter (pCAT) and the methanol oxidase promoter (pMOX) are comparable on methanol, but the maximum expression level of the pCAT is reached more than 24 h earlier. By varying the terminators, a 6-fold difference in gene expression was achieved with the MOX terminator boosting gene expression on all carbon sources by around 50% compared to the second-strongest terminator. It was shown that this exceptional increase in gene expression is achieved by the MOX terminator stabilizing the mRNA, which results in an increased transcript level in the cells. We further found that different pairing of promoters and terminators or the expression of a different gene (β-galactosidase gene) did not influence the performance of the genetic parts. Consequently, it is possible to mix and match promoters and terminators as independent elements to tune gene expression in *O. polymorpha*.

## Introduction

The yeast *Ogataea polymorpha* (formerly *Hansenula polymorpha*) has been extensively applied as a superior protein factory for pharmaceuticals such as hepatitis B vaccines (Janowicz et al., 1990; Seo et al., 2008) and insulin-like growth factors (Faber et al., 1996). However, *O. polymorpha* also bears potential as an excellent production platform for industrially-relevant bio-based chemicals. The yeast can tolerate high temperatures of up to 50°C (Cabeç-Silva and Madeira-Lopes, 1984), grows in a wide pH range from 2.5 to 6.5, is generally regarded as safe (GRAS status) and several Ogataea species have been sequenced (Manfrão-Netto et al., 2019). *O. polymorpha* can not only grow on interesting carbon and nitrogen sources like xylose (Ryabova et al., 2003; Martínez-Cartas et al., 2019) and glycerol (de Koning et al., 1987) but it is also one of few yeasts that can utilize methanol as the sole carbon and energy source (de Koning et al., 1987). Especially the latter, rare ability strengthens its upcoming role as an industrial workhorse.

Methanol can be produced sustainably from CO_2_ and green hydrogen and has recently gained much public attention as microbial feedstock (Liebal et al., 2018; Cotton et al., 2020; Fabarius et al., 2021). Despite *O. polymorpha*’s obvious potential as a host organism for sustainable bioproduction processes, metabolic engineering tools for this yeast are still rare. There are only some tools publicly available for advanced metabolic engineering of *O. polymorpha* (Manfrão-Netto et al., 2019). Recently, CRISPR/Cas9 based gene editing strategies were established for *O. polymorpha* (Numamoto et al., 2017; Juergens et al., 2018; Wang et al., 2018; Gao et al., 2021), which could enable a huge breakthrough in metabolic engineering in the years to come. Despite these advances, studies about the production of industrially relevant chemicals from methanol with *O. polymorpha* are scarce. Recently, Zhai et al. (2021) demonstrated the production of a fatty alcohol in *O. polymorpha* using methanol as carbon source and applying the CRISPR/Cas9 system. However, other metabolic engineering tools, including well-characterized genetic elements like promoters, terminators, and integration sites are still scarce and urgently needed to match the diversity of tools available for the model organism *Saccharomyces cerevisiae*. Promoters primarily control the expression strength and pattern of genes in a recombinant host. For metabolic engineering of yeasts, usually, endogenous promoters are used, which exhibit high activity in the host but are also subject to transcriptional regulation as a response to environmental signals, e.g., carbon or nitrogen sources (Peng et al., 2015).

*O. polymorpha* is well known for its strong, often methanol inducible, native promoters. Especially the promoter of the methanol oxidase (pMOX) has been studied profoundly (Gellissen et al., 2005; Wagner and Alper, 2016; Manfrão-Netto et al., 2019). At large, promoters of the methanol pathway are repressed in the presence of glucose, derepressed on glycerol and activated on methanol in *O. polymorpha* (Hartner and Glieder, 2006). Regulatory promoter stretches were identified in front of the pMOX promoter. Two upstream activation sequences (UAS) and one upstream repressing sequence (URS) are located in the pMOX promoter region (Gödecke et al., 1994). In a study by Suppi et al. (2013) glucose 6-phosphate was proposed to be a signaling molecule for repression as the pMOX promoter was only repressed when glucose was present in the cell in its phosphorylated form. Even though other studies have examined the strength of *O. polymorpha*’*s* native promoters before (Hartner and Glieder, 2006; Zhai et al., 2021), there is no study closely examining their temporal expression pattern nor the effects of combining the characterized promoters with different terminators.

In contrast to promoters, terminators have been paid very little attention in most eukaryotes. Terminators determine the secondary structure, length, and polyadenylation of the mRNA’s 3′ untranslated region (3′UTR). It has been demonstrated that terminators can increase gene expression through enhanced mRNA stability, thereby improving protein production. These differences in mRNA stability were frequently traced back to differences in the secondary structure, GC-content, and length of the 3′UTR (Tanguay and Gallie, 1996; Imamachi et al., 2017; Ito et al., 2020). Further, it has been shown that an intact polyadenylation signal is essential for proper transcription termination and mRNA stability, as degradation of an mRNA is dependent on the length of the poly(A)-tail (Connelly and Manley, 1988; Eckmann et al., 2011; Tian and Graber, 2012). Even though several studies have shown a major influence of terminators on gene expression in different yeast species (Curran et al., 2015; Wei et al., 2017; Ramakrishnan et al., 2020), heterologous gene expression in *O. polymorpha* still relies on a very limited number of standard terminators, which have not been characterized systematically.

Here, we followed the expression of a green fluorescent protein (GFP) as a reporter gene for promoter and terminator activities. The chosen GFP variant, ubiM-GFP, has a drastically reduced half-life of 1.5 h *in vivo* (>36 h for a GFP WT variant) (Reider Apel et al., 2017). This GFP variant enables time-resolved monitoring of gene expression throughout a cultivation. Using the ubiM-GFP, maximum promoter and terminator activities were determined for cultivations on glucose, glycerol and methanol. Further, the time point was determined at which the maximal expression level was reached. Substrate mixes of glucose or glycerol with methanol were tested for their effect on the promoter performance during the cultivation. Furthermore, we examined the impact of combining promoters of varying strength with different terminators in *O. polymorpha.* Transcript stability, influenced by the secondary structure and the GC-content of the 3′UTR, was found to be a major factor in determining terminator strength. Although previous studies have compared some promoters’ relative strengths on glucose and methanol as primary substrates, the promoter activity and especially the terminator activity throughout batch cultivations has not been examined in *O. polymorpha*. This study provides a toolbox for optimizing gene expression during batch cultivations and on different carbon sources that will foster the utilization *O. polymorpha* as chassis for bioproduction processes.

## Materials and Methods

### Strains and Media

All created yeast strains in this project are based on *Ogataea polymorpha* NCYC495 *leu1.1,* whose genome has been sequenced (Riley et al., 2016). To facilitate genomic integration, the Δyku80 variant of this strain, deficient in non-homologous end joining, was used (Saraya et al., 2012). For pre-cultures, the yeast cells were grown at 37°C in a rich YEP medium (1% yeast extract, 2% peptone, 2% glucose), supplemented as required with 100 µg/ml zeocin, 200 µg/ml hygromycin or 30 µg/ml leucine. For growth analysis and fluorescence screenings during microtiter plate cultivation (see section *BioLector cultivation*), the defined mineral Verduyn medium (Verduyn et al., 1992) was used, supplemented with either 15 g/L glucose, 15 g/L methanol or 15 g/L glycerol as carbon sources. The equal concentrations for each substrate (15 g/L) were chosen to keep the C-mol amount constant (with a maximum difference of 10% between the substrates). For the carbon mix experiments 10 g/L methanol was blended with 5 g/L glucose or glycerol. Complete substrate consumption was verified for the wildtype via HPLC (Supplementary Figures S4, S5). Competent *E. coli* cells (NEB^®^ 10-beta, High Efficiency) were used for recombinant plasmid preparation. *E. coli* was grown on lysogeny broth (LB) containing 100 µg/ml ampicillin at 37°C.

### Plasmid Construction

All plasmids used in this study are based on the pHIP expression vectors for *O. polymorpha* (Saraya et al., 2012; https://www.rug.nl/research/molecular-cell-biology/research/the-hansenula-polymorpha-expression-system?lang=en *)* and are listed in the supplementary information Supplementary Table S1. All primers were synthesized by Eurofins Genomics (Ebersberg, Germany) and are listed in Supplementary Table S2. Genetic parts for plasmid assembly were amplified using Q5^®^High-Fidelity Polymerase [New England Biolabs (NEB); Ipswich, MA, United States] and the assembly of the amplicons was performed *in vitro* using the NEBuilder^®^ HiFi DNA Assembly (NEB).

For the expression analysis of both promoters and terminators, a short half-life green fluorescence protein, ubiM-GFP, was cloned into pHIP plasmids. Here, an ubiquitin degradation tag is fused to the 5′-end of the GFP gene. This fusion shortens the half-life of the protein and thus makes it possible to follow the dynamics of GFP expression throughout a cultivation (Reider Apel et al., 2017). Several constitutive and inducible promoters were chosen from the pHIP plasmid collection for expression analysis. All promoter sequences are listed in Supplementary Table S3. For the terminator study, novel and established terminators were characterized. For novel terminators from *O. polymorpha,* a length of 250 base pairs downstream of the stop-codon was chosen. The terminator sequences were cloned at the 3′-end of the GFP reporter gene under the control of the pCAT promoter. All terminators and their sources are listed in Supplementary Table S4.

### Genomic Integration

For genomic integration into *O. polymorpha* the expression vectors were linearized at unique restriction sites in their promoter region, allowing site-specific integration in the exact promoter region of the *O. polymorpha* genome. Transformation was performed through LiAc/single-stranded carrier DNA/PEG transformation as described by (Holkenbrink et al., 2018). After transformation, the cells were spread on YEP agar plates with the appropriate selective medium and incubated for 2–3 days at 37°C. Correct integration was verified through colony-PCR using the Phire Plant Direct PCR Master Mix (Thermo Fisher Scientific, Waltham, MA, United States). For all colony-PCR reactions, one primer binds on the integrated cassette and the other one on the surrounding genomic region. Quantitative real-time (qPCR) was applied to verify that only a single copy of the respective DNA cassette was integrated into each strain (Supplementary Figures S1, S2). Correct integration was further verified by whole-genome sequencing of three exemplary strains (data not shown).

### BioLector Cultivation

To analyze the created mutant strains for growth and GFP fluorescence, the microtiter plate cultivation system BioLector was applied (Samorski et al., 2005; commercialized by m2p-labs GmbH, Aachen, Germany). The BioLector combines the advantage of online measuring techniques (GFP measurements and scattered light measurements) and optimized process parameters (Funke et al., 2010). Especially the oxygen transfer rate is a crucial parameter for process control which is fully optimized in the BioLector by high shaking frequencies and an optimized shape of the well (FlowerPlates^®^). The possibility of a direct scale-up from the BioLector to a fermenter was described for *O. polymorpha* (Kensy et al., 2009). All BioLector cultivations were performed in 48 well FlowerPlates^®^ with a clear bottom and were covered with an adhesive gas-permeable membrane (m2p-labs GmbH). Each well was filled with 1 ml Verduyn and inoculated to an initial optical density at 600 nm (OD 600 nm) of 0.2. Experiments were performed in biological triplicates. Biomass accumulation was monitored online by measuring the scattered light in each well at 620 nm (Gain 40), while GFP fluorescence was measured with a 488 nm excitation filter and a 520 nm emission filter (Gain 100). The measured GFP fluorescence was then normalized to the biomass density in the culture (i.e., scattered light value at 660 nm).

### β-galactosidase Activity Assay

The β-galactosidase assay was performed in biological and technical triplicates in a 96-well plate format using a yeast β-galactosidase assay kit (Thermo Scientific™, Waltham, MA, United States). Cultures were grown for 72 h on Verduyn medium with methanol as a carbon source. The β-galactosidase activities were normalized to the optical density measured at 660 nm.

### Determination of mRNA Abundance and Stability

To analyze mRNA abundance and transcript stability, the total RNA was isolated from *O. polymorpha* cultures grown on methanol. To this end, transcription was inhibited in a culture of exponentially growing *O. polymorpha* cells by adding 100 µg/ml 1,10-phenanthroline (Sigma-Aldrich, St. Louis, MO, United States). After adding the antibiotic, 1 ml of culture was harvested at several time points for 1 h. In order to enable a higher time resolution, we focused on sampling as often as possible instead of having biological triplicates, as growth of the used strains was reproducible in all of our experiments. The harvested cells were pelleted and frozen in liquid nitrogen. Cells were then lysed mechanically using ZR bashing Bead Lysis Tubes (2 mm) (Zymo Research, Freiburg, Germany). After lysis, the cells were briefly cooled on ice and then the RNA was extracted with the Monarch Total RNA Miniprep Kit (NEB), following the manufacturer’s instruction for *Tough-to-Lyse Samples*.

Genomic DNA contaminations in the samples were removed by treating the extracted RNA with the Invitrogen™ TURBO DNA-*free*™ Kit (Thermo Fisher Scientific, Waltham, MA, United States). The mRNAs in the samples were converted into cDNA with the ProtoScript^®^ II First Strand cDNA Synthesis Kit (NEB) using the supplied Oligo-dT primers. Subsequent quantification was performed by qPCR, using the Luna Universal qPCR Master Mix (NEB). As a reference gene in *O. polymorpha* TAF10 (Hanpo2_11508) was chosen. To determine the relative abundance of the target mRNAs in the cells, the amount of target transcript was normalized to the amount of reference transcript (TAF10) at each time point of the experiment. To evaluate the stability of the target constructs the 2^–ΔΔCt^ method was applied (Livak and Schmittgen, 2001). In brief, the initial sample taken before transcription inhibition (*t* = 0) was used as a calibrator sample. For this sample, the difference of Ct-values between target and reference transcript was calculated and set to 1. Then the differences between the Ct-values at all other time points of the experiment were subtracted from this value of the calibrator sample. It has to be considered that in this experiment, the mRNA of the reference construct also decays, with a rate that is assumed to be constant in all tested strains. Therefore, the calculated values for the relative transcript decay do not show the actual decay of the transcript but only allow for a relative comparison of the samples among each other.

### *In Silico* Analysis of 3′-UTRs.

The minimal free energy (MFE) was taken as a measure for the stability of the secondary structure of the 3′UTR. The MFE for the secondary structure of all 3′UTRs was calculated using the RNAfold program of the ViennaRNA package 2.0 (Lorenz et al., 2011). As the MFE of a sequence is heavily dependent on its length, sequences of fixed lengths (20–120 nt) were extracted from the 3′UTR of all terminator constructs for analysis. At the same time, the GC-content of those fixed-length sequences was calculated. The Spearman’s correlation coefficient (Spearman’s Rho) was calculated to identify correlations between the MFE or the GC content and the GFP expression level of the terminator strains.

## Results and Discussion

### The Carbon Source Strongly Influences Promoter Activity

The influence of *O. polymorpha*’s promoters on gene expression during batch cultivations was evaluated by using them to express the reporter gene ubiM-GFP (Reider Apel et al., 2017). The GFP gene was put under the control of native promoters from *O. polymorpha*. Here, we comprehensively characterized different methanol inducible and constitutive promoters. With this selection of promoters we aim to cover a broad range of activities on different substrates. As methanol inducible promoters, the methanol oxidase promoter pMOX, the promoter of the catalase pCAT and the promoter of the dihydroxyacetone synthase pDHAS, were analyzed. Furthermore, we chose the constitutive promoters pTEF1 and pTEF2, of the translation elongation factors 1 and 2 and the alcohol dehydrogenase promoter, pADH1. Detailed sequence information is provided in Supplementary Table S3. The promoter strength was characterized using the GFP fluorescence signal normalized to the scattered light signal (indicator for biomass) measured over the entire batch cultivation in the microbioreactor device BioLector. The terminator tAMO was used as a standard for all constructs as it is widely applied, e.g., in the pHIP plasmid system.

**FIGURE 1:**
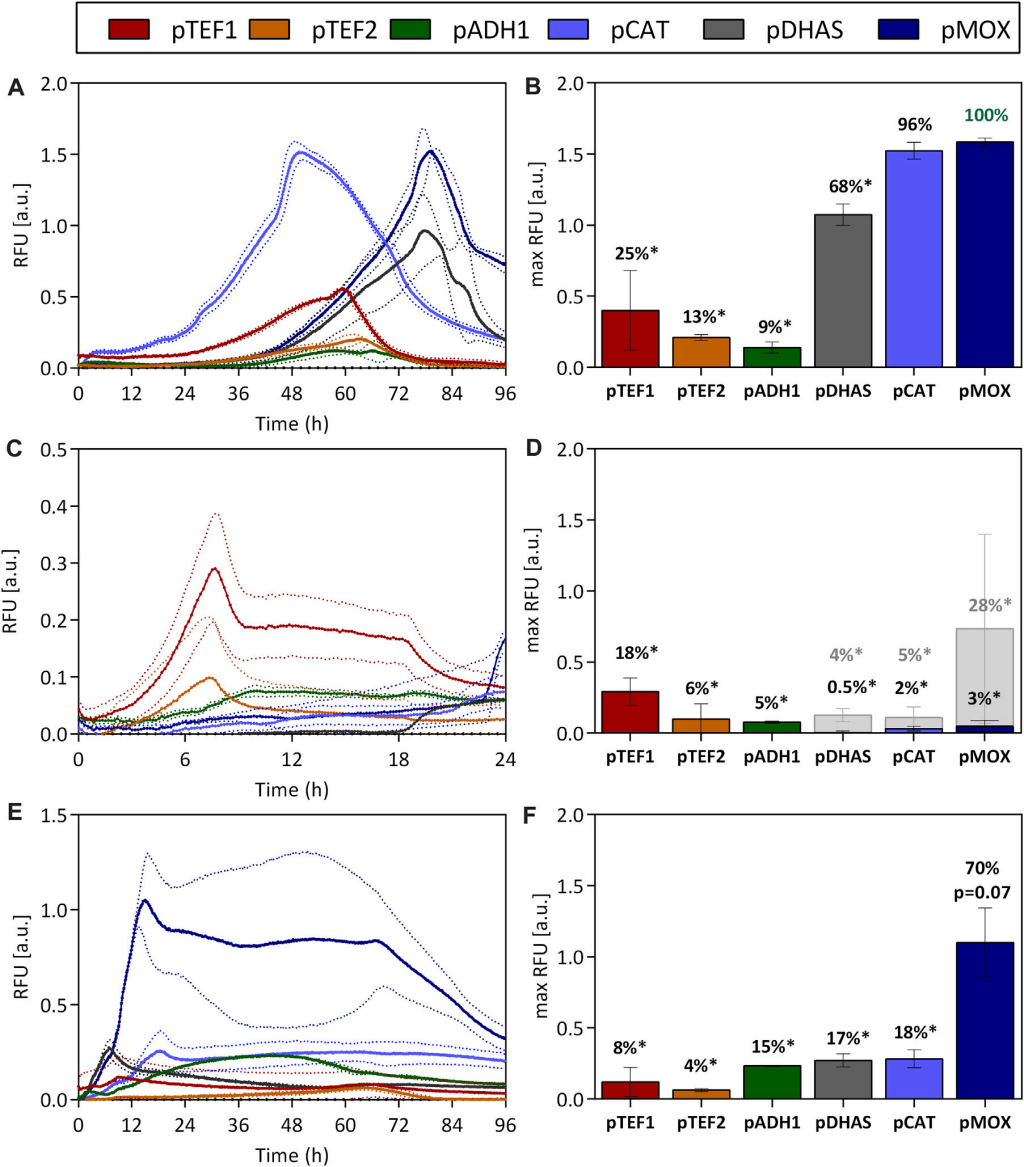
Promoter activities of *O. polymorphas*’ native promoters when utilizing methanol **(A,B)**, glucose **(C,D)**, and glycerol **(E,F)** as carbon sources. The influence of *O. polymorpha*’s promoters on gene expression was evaluated by using them to express the reporter gene ubiM-GFP. The GFP gene was put under the control of native promoters from *O. polymorpha*. The two constitutive promoters pTEF1 and pTEF2, pADH1, and promoters involved in methanol metabolism pDHAS, pCAT, and pMOX are characterized. The GFP’s fluorescence was normalized to the biomass density (expressed as RFU) and measured over the time-course of the cultivation in 48-well plates. The fluorescence and growth dynamics during the cultivation are indicated in **(A,C,E)** while maximum fluorescence signals and percentages correlated to the highest fluorescence maximum [pMOX 100% in **(B)**] are shown in **(B,D,F)**. Statistical significance (indicated with * when *p* < 0.05) was tested with an unpaired *t*-test between the max RFU of the various promoters and the highest max RFU of the dataset [pMOX 100% in green in **(B)**] see Supplementary Table S5. Dotted lines **(A,C,E)** and error bars **(B,D,F)** represent the standard deviation from three biological replicates. Light grey bars indicated in **(D)** represent maximum RFU after glucose depletion.

During the cultivation of *O. polymorpha* on methanol, the pMOX promoter showed the highest fluorescence signal. Under these conditions, a maximum fluorescence relative to the scattered light signal (RFU) of 1.58 ± 0.03 was reached (Figures 1A,B). For easy comparison between the promoters, this was set to 100% (Figure 1B). With 1.52 ± 0.06 RFU the pCAT promoter obtained 96% of the pMOX activity and is therefore just as strong on methanol. An intermediate expression strength was observed for pDHAS, which reached a maximum GFP level of 1.07 ± 0.07 RFU, 68% of the pMOX activity (Figures 1A,B). Even though GFP fluorescence is also impacted by differences in growth, especially the lag phase length, it is striking that the pCAT strain always reaches maximum fluorescence earlier than pMOX and pDHAS strains. Using the pCAT, maximum GFP fluorescence was reached after 50 h, while full GFP expression with pMOX and pDHAS was obtained after 80 h of growth (Figure 1A). During methanol assimilation, H_2_O_2_ is formed, which the catalase converts to H_2_O and O_2_. Therefore, the early induction of pCAT on methanol could be a safety mechanism to prevent the accumulation of the toxic compound produced during methanol assimilation. All other tested promoters showed an expression strength below 25% of the pMOX on methanol (Figures 1A,B).

On glucose (Figures 1C,D), the highest GFP expression was reached with the constitutive promoter pTEF1, which generated a fluorescence signal of 0.29 ± 0.10 RFU reaching only 18% of the maximum expression obtained with the pMOX promoter on methanol. TEF1 promoters are some of the most well-known strong yeast promoters and are therefore utilized across species in many production processes. However, this drastic difference in expression strength between pTEF1 on glucose and pMOX on methanol indicates the vast potential of the pMOX promoter for bio-production when using methanol as a carbon source. On glucose, all promoters other than the pTEF1 promoter had only low activities below 6%. Nevertheless, when glucose is limited after 18 h of cultivation (Supplementary Figure S5), the pMOX promoter is induced and obtained 28% of its activity on methanol (Figures 1C,D). Also, pCAT and pDHAS indicated an induction by glucose depletion but only to a much lower extent (Figures 1C,D).

Compared to glucose, promoter activities were higher for all promoters involved in methanol metabolism when *O. polymorpha* was cultivated on glycerol (Figures 1E,F). This effect is known as de-repression (Hartner and Glieder, 2006). The pMOX promoter generated a signal of 1.10 ± 0.24 RFU, which is 70% of its activity on methanol. The pCAT promoter, which is equally strong on methanol (Figures 1A,B), only reached a maximum of 0.28 ± 0.06 RFU on glycerol, which is only 18%, of the activity of the pMOX on methanol (Figures 1E,F). Compared to methanol, maximum promoter activities are already obtained after 10–15 h due to a superior growth on this carbon source. Thus, cultivations utilizing glycerol as carbon sources have the advantage that they enable fast growth and comparatively strong promoter activity, especially for the pMOX promoter. This is a huge advantage for many production processes that do not want to deal with methanol, which is explosive and toxic, requiring additional safety measures. Production processes with the closely related yeast *Pichia pastoris* cannot use the native methanol oxidase promoter (pAOX1) for productions on glycerol as it is repressed on this carbon source (Hartner and Glieder, 2006). Nevertheless, maximal promoter activity is only obtained when *O. polymorpha* is cultivated on methanol, which empowers both the pMOX and the pCAT to exceptionally high activity.

### Induction With a Glycerol-Methanol Substrate Mix

**FIGURE 2:**
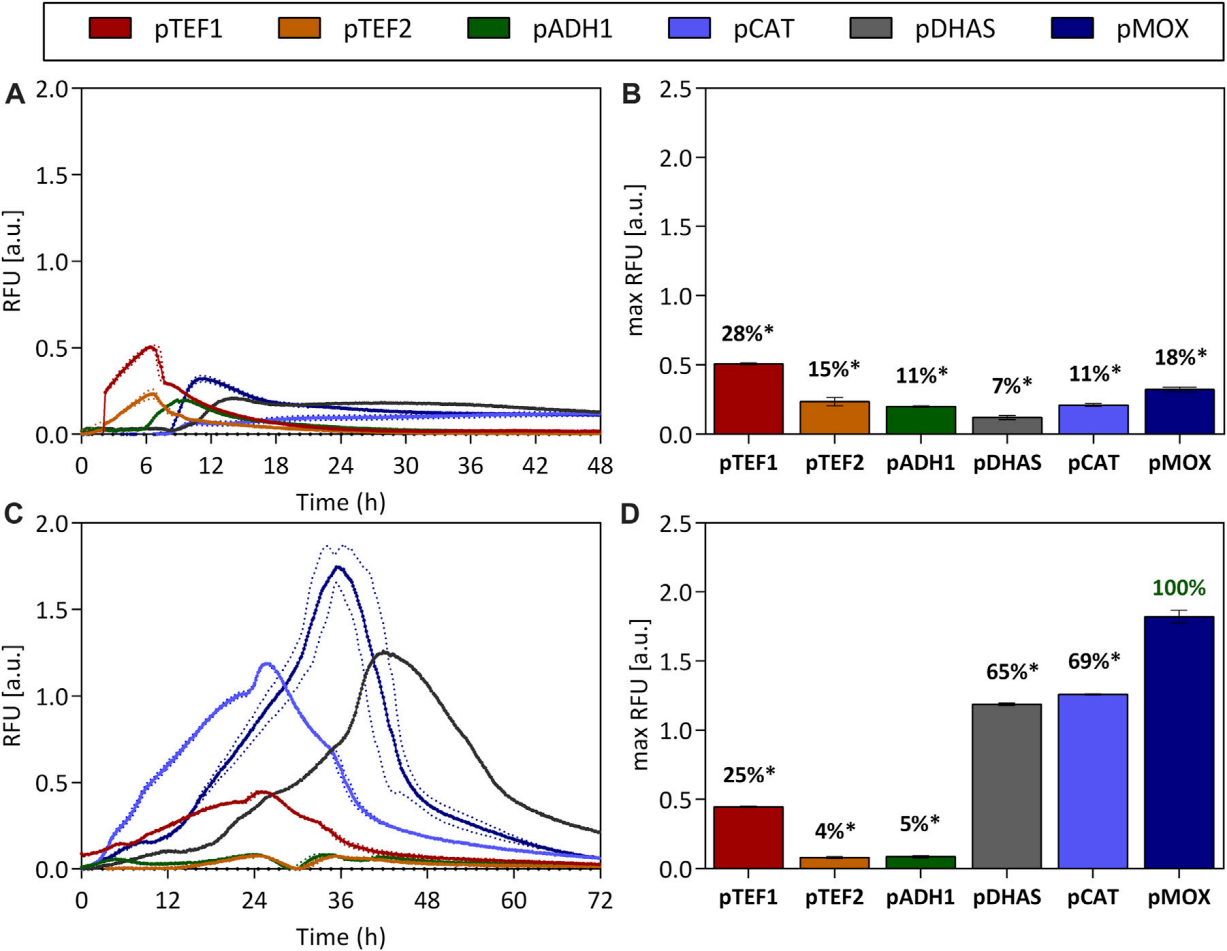
Promoter activities on the substrate mixes glucose-methanol **(A,B)** and glycerol-methanol **(C,D)**. The constitutive promoters pTEF1 and pTEF2, pADH1 and promoters implicated in methanol metabolism such as pMOX, pDHAS and pCAT were tested over the time-course of a batch cultivation **(A,C)** on the two substrate mixes. The GFP’s fluorescence normalized by the biomass density (expressed as RFU) was measured in a 48-well plate format. Additionally, maximum RFU values were extracted and percentages were calculated in relation to the strongest promoter pMOX on glycerol-methanol (100%). Statistical significance (indicated with *when *p* < 0.05) was tested with an unpaired *t*-test between the max RFU of the various promoters and the highest max RFU of the dataset [pMOX 100% in green in **(D)**] see Supplementary Table S6. Dotted lines **(A,C)** and error bars **(B,D)** represent the standard deviation from three biological replicates.

Growth rates of *O. polymorpha* on glucose and glycerol are with 0.53 1/h and 0.24 1/h (Supplementary Figure S3) considerably higher than growth rates on methanol (0.13 1/h Supplementary Figure S3), making glucose and glycerol attractive carbon sources. However, full induction of the most potent promoter is only obtained with methanol. Therefore, the effect of substrate mixes of glucose and methanol (Figures 2A,B) and glycerol and methanol (Figures 2C,D) on promoter activity was tested during batch cultivation. On a glucose-methanol mix, only a low promoter activity with a maximum of 28% for the pTEF1 promoter (0.49 ± 0.03 RFU), was observed after 6 h (Figures 2A,B). Yet, when glucose was depleted after 8 h, pDHAS, pCAT and pMOX were induced by 7%, 11% and 18%, respectively (Figure 2B). Here, pMOX showed a maximum GFP fluorescence of 0.32 ± 0.02 RFU after around 10 h (Figure 2A). No full induction of pDHAS, pCAT or pMOX was observed with a glucose-methanol substrate mix. In contrast, when *O. polymorpha* was cultivated on a glycerol-methanol substrate mix (Figure 2B), pMOX showed a maximum GFP level of 1.71 ± 0.16 RFU, comparable to its activity on pure methanol as the carbon source (1.58 ± 0.03 RFU in Figures 1A,B). Also, pDHAS indicated a similar induction of 1.19 ± 0.01 RFU and 69% of the activity on methanol (1.07 ± 0.07 RFU, 68% in Figures 1A,B). However, the pCAT promoter, which is equally strong as pMOX on pure methanol, was not fully induced by a substrate mix of glycerol-methanol. pCAT obtained 1.19 ± 0.01 RFU, which is only 65% of the pMOX (Figures 2C,D) and considerably lower than on pure methanol (Figure 1B). Furthermore, on the glycerol-methanol mixture, we observed that the pCAT promoter reached its maximum expression level approximately 10 h earlier than the pMOX and 15 h earlier than the pDHAS (Figure 2C). This trend was also observed on pure methanol (Figure 1A). However, it is less pronounced on the mixture due to the reduced activity of pCAT on glycerol. To sum up, no full induction of the promoters involved in methanol utilization is feasible on a glucose-methanol mix, while induction of pMOX and pDHAS and to a lower extent for the pCAT could be obtained with a mixture of methanol and glycerol. Hence, a production process that requires strong induction and fast growth using a mixture of glycerol and methanol is an attractive option.

### Characterization of Native and Heterologous Terminators

In line with the promoter characterization, the influence of terminators on gene expression was evaluated by using them to express the ubiM-GFP reporter gene. The GFP gene was put under the control of the pCAT promoter, and the terminator sequences were cloned seamlessly downstream of the stop codon. The terminator sequences in this experiment (Supplementary Table S4) were chosen so that they are expected to boost the expression of the ubiM-GFP gene in *O. polymorpha*. The terminators of the *O. polymorpha* methanol oxidase (MOX), catalase (CAT), and formate dehydrogenase (FMD) were selected as the corresponding genes are reported to show exceptionally high expression upon growth on methanol (van Zutphen et al., 2010). In the methylotrophic yeast *Pichia pastoris*, several terminators have already been characterized for their impact on gene expression (Prielhofer et al., 2017). Therefore, homologs of strong terminators from *Pichia pastoris* were identified in *O. polymorpha* using a BLAST search and included in this set (e.g., the terminators of the glyceraldehyde-3-phosphate dehydrogenase (tTDH3) and the protein compound 2A of the 40S ribosome subunit (tRPS2A). Further, terminators from genes with a high constitutive expression in *O. polymorpha* were included in the set, such as the terminator of the plasma membrane H^+^-ATPase (tPMA1) (Kang and Gellissen, 2005; van Zutphen et al., 2010). Additionally, heterologous terminators from other yeast species (i.e., tTEF2 from *S. cerevisiae,* tTEF1 from *Ashbya gossypii,* and tAOX1 from *Pichia pastoris)* were included as they are routinely used to control gene expression in various yeasts (Sugimoto et al., 2009; Guo et al., 2015; Ramakrishnan et al., 2020). Together, these chosen terminators make up a set of homologous and heterologous sequences from either constitutively expressed or methanol-inducible genes with a reported high expression level.

**FIGURE 3:**
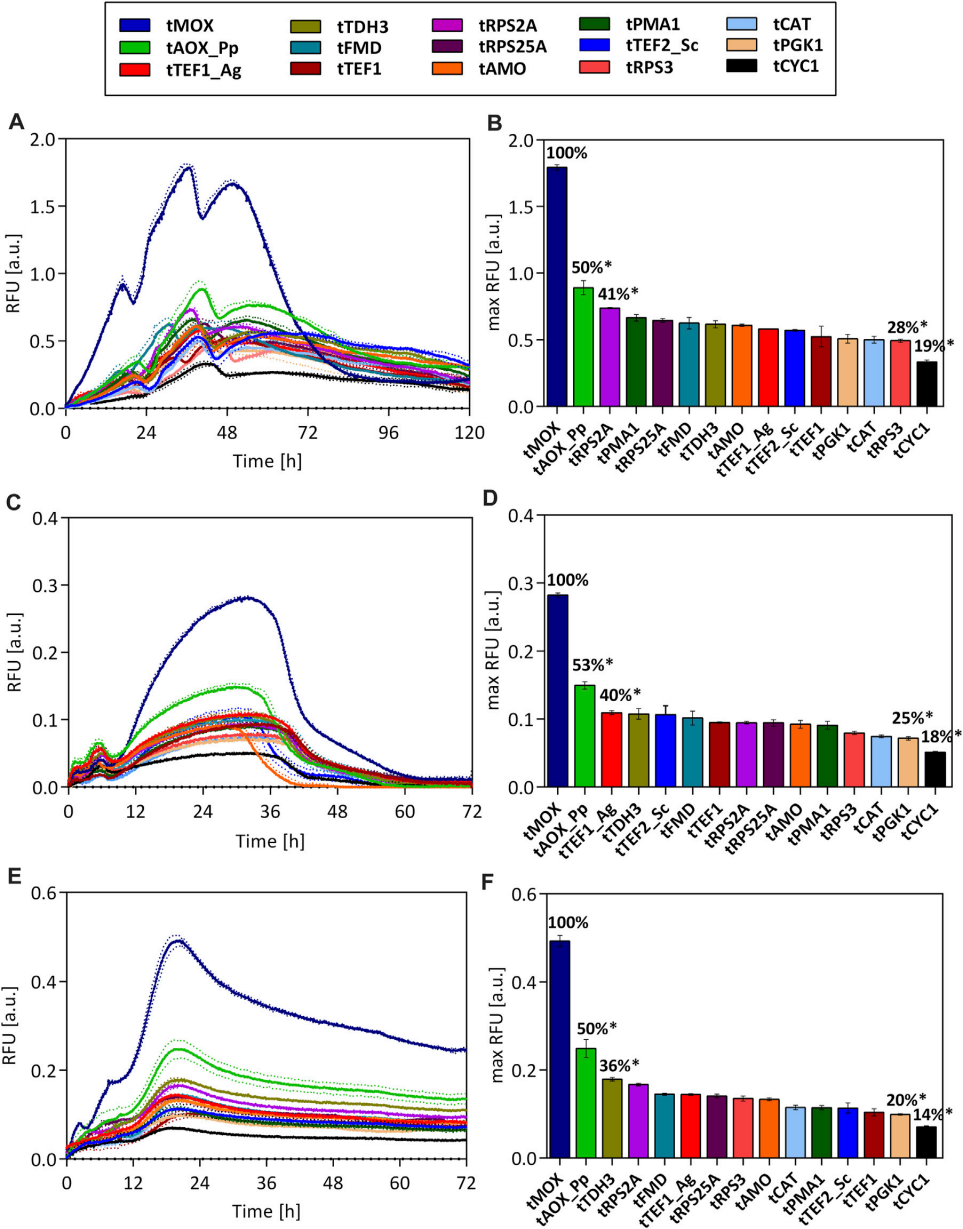
GFP fluorescence throughout the cultivation of *O. polymorpha* strains. Expression of an ubiM-GFP gene under the control of the pCAT promoter and varying terminators on methanol **(A)**, glucose **(C)**, and glycerol **(E)** as carbon source. Maximal GFP fluorescence for each strain on methanol **(B)**, glucose **(D)**, and glycerol **(F)**. All measured fluorescence values were normalized to the biomass density (scattered light value) in the culture. Dotted lines **(A,C,E)** and error bars **(B,D,F)** represent the standard deviation from three biological replicates. Statistical significance (indicated with *when *p* < 0.05) was tested with an unpaired, two-tailed *t*-test between the max RFU of the various terminator strains and the highest max RFU of the tMOX strain on each carbon source (tMOX 100%) Supplementary Table S7.

GFP expression for all terminator strains was measured in microtiter plate cultivations with glucose, glycerol, and methanol as carbon sources. All measured GFP signals were normalized to the biomass density in the cultures, i.e., the scattered light value (Figure 3).

Independent of the used carbon source, drastic differences in the GFP signal were observed between the tested strains. On all carbon sources, the strain carrying the terminator from the methanol oxidase (tMOX) of *O. polymorpha* showed by far the strongest GFP signal (max. RFU on methanol: 1.79 ± 0.02). Regardless of the carbon source selected, the GFP expression in the tMOX strain is significantly elevated compared to the strain with the AOX1 terminator from *Pichia pastoris* (tAOX1_Pp), which showed the second highest GFP signal (cp. Figure 3; Calculations in Supplementary Information). However, the tAOX_Pp terminator still results in (50%–53%) of the maximum expression observed for the tMOX strain. This indicates that applying heterologous terminators can also result in high gene expression in *O. polymorpha*. Heterologous terminators pose useful tools in metabolic engineering as they reduce the risk of unwanted integration or looping-out of genetic constructs due to recombination with homologous regions of the host genome (MacPherson and Saka, 2017). Apart from the tMOX and the tAOX_Pp, several novel terminators were identified (e.g., tTDH3, tRPS2A and tRPS25A), that led to comparable expression levels as commonly used terminators such as the tAMO or the tTEF1_Ag.

When looking at the temporal GFP expression pattern, there are no major differences between the tested strains regarding the time point of maximal expression. This suggests that the terminators do not play a role in determining when a gene is expressed. Instead, the time point of expression is primarily dictated by the chosen promoter.

It is striking that the relative differences in the GFP signal between the tested strains remain similar during growth on methanol, glycerol, and glucose. For all three carbon sources, the highest GFP signal was measured for the strain carrying the tMOX terminator, followed by the strain with the tAOX_Pp terminator, for which the maximum GFP signal always corresponded to 50%–53% of the maximum determined for the tMOX strain (Figures 3B–F). The lowest GFP fluorescence was always measured for the strain with the tCYC1 terminator (16%–18% of the tMOX signal). The strains with the remaining terminators consistently showed intermediate fluorescence levels, ranging between 20% and 41% of the tMOX strain. Terminators from genes upregulated on methanol (tMOX, tCAT and tFMD) did not result in a higher expression on methanol, compared to glucose and glycerol. Instead, the tMOX strain, produced by far the strongest GFP signal on glucose, relative to the other terminators. Therefore, these results indicate that in *O. polymorpha* the effect of the terminator on gene expression is nearly independent of the carbon source used. The results also demonstrate that the exceptionally high expression level of the MOX gene (Gödecke et al., 1994; van Zutphen et al., 2010), can also be attributed to its terminator. However, not all genes involved in the methanol assimilation pathway of *O. polymorpha* have a strong terminator. The terminators of the formate dehydrogenase (tFMD) and the catalase (tCAT), only resulted in intermediate GFP expression, even though the corresponding genes exhibit an expression strength on methanol comparable to the MOX (van Zutphen et al., 2010). Thus, it can be concluded that highly expressed genes or genes having a strong promoter do not necessarily have an equally strong terminator in *O. polymorpha*.

Looking at the absolute values of GFP fluorescence on all carbon sources (Figures 3A–E), the strongest expression level was measured on methanol (max RFU for tMOX strain 1.79 ± 0.02), while on glycerol and glucose, the expression level is significantly lower (max RFU tMOX glycerol: 0.49 ± 0.01; glucose 0.28 ± 0.003). These differences in the absolute expression level are dictated by the used promoter pCAT, which showed precisely this gene expression pattern on the different carbon sources (Figure 1). This behavior corresponds to observations made for *S. cerevisiae*, where the culture conditions (e.g., carbon source) have a higher impact on promoter activity than on terminator activity (Ito et al., 2013).

Considering all tested strains, a roughly 6-fold difference in the GFP expression level can be achieved on all carbon sources by varying the terminators alone. Consequently, these terminators represent valuable tools to tune gene expression in *O. polymorpha* for metabolic engineering applications. Further, it should be considered that only 15 terminators from genes with a high expression level were chosen in this study. A genome-wide analysis of terminator regions in *S. cerevisiae* found a 70-fold difference in their activity (Yamanishi et al., 2013). Thus, it is possible that characterizing additional *O. polymorpha* terminators will result in an even broader range of expression levels, which could extend the terminator toolbox for *O. polymorpha*.

### The Effect of Terminators on mRNA Abundance and Stability

**FIGURE 4:**
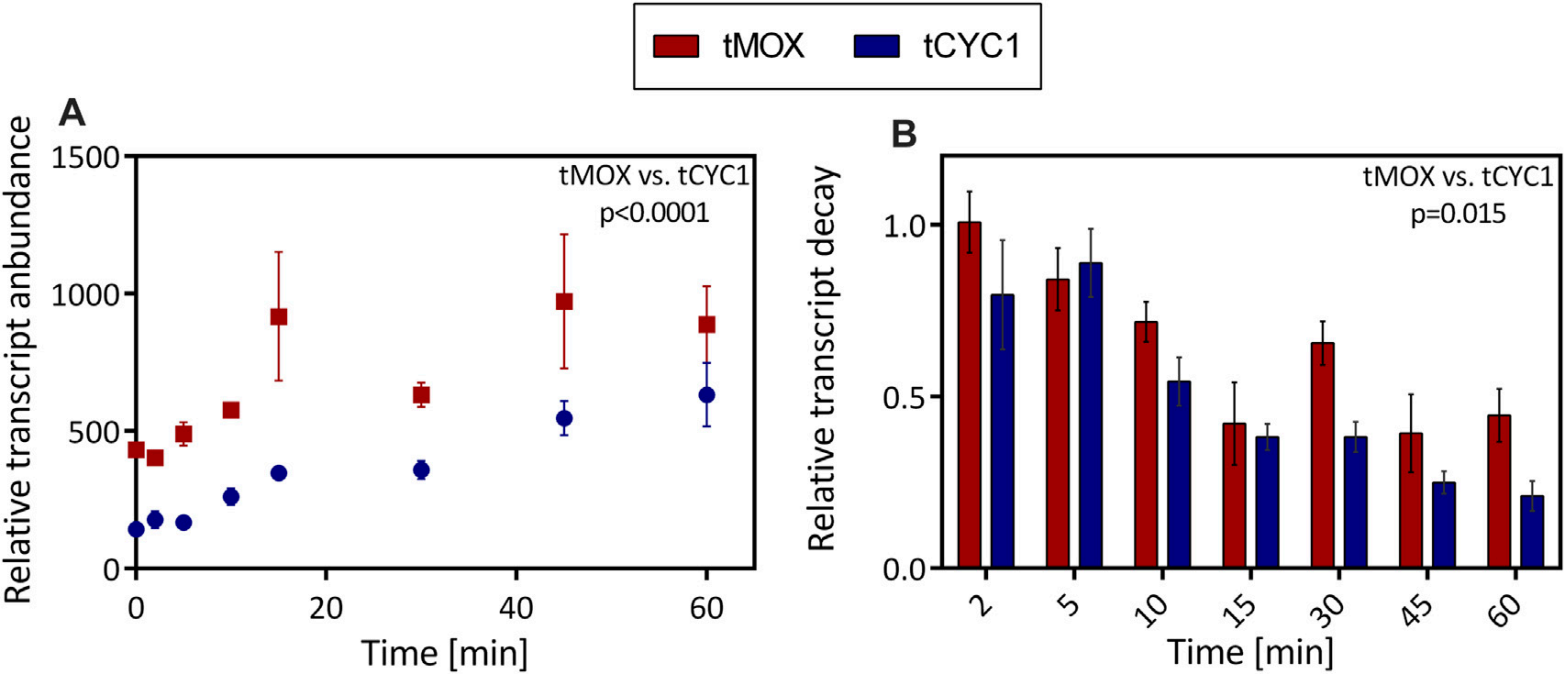
Comparison of mRNA abundance and decay in *O. polymorpha* strains expressing a genetic construct with the tMOX or tCYC1 terminator. In exponentially growing cultures, transcription was inhibited through the addition of the antibiotic 1,10-phenanthroline. After inhibition, RNA was extracted at regular time points within 1 h. **(A)** Level of target mRNA (tMOX and tCYC1) relative to the mRNA of the reference gene TAF10. **(B)** Relative transcript decay: Abundance of the target mRNAs relative to the reference gene TAF10 and the initial difference between target and reference mRNA at *t* = 0. Error bars represent three technical replicates. The means transcript abundance **(A)** and the relative transcript decay **(B)** were compared using a paired, two-tailed Student’s *t*-test. Statistical significance is indicated as the *p* value on the graph (Caluclations in Supplementary Information).

Having seen that the choice of a terminator can dramatically influence the level of gene expression in *O. polymorpha*, the mechanism underlying these differences was examined further. Therefore, transcript abundance and degradation were analyzed in the strains with the tMOX and the tCYC1 terminator constructs. These constructs led to the highest (tMOX) and the lowest (tCYC1) GFP expression in the preceding terminator characterization (Figure 3). In these two strains, transcription was inhibited, and the total RNA was extracted from the cultures. The level of target mRNA was afterward determined through qPCR analysis and normalized to the reference gene TAF10. Figure 4A shows the amount of transcript relative to the reference gene for the tMOX and the tCYC1 strain. To assess statistical significance of the results, the means of the transcript abundance were compared between the tMOX and tCYC1 strain, using a paired, two-tailed Student’s t-test. Further, the differences of the means at the individual measuring time points were compared between the two strains with an unpaired, two-tailed Student’s t-test. All calculations can be found in the Supplementary Information.

At all time points of the experiment, except 60 min after transcription inhibition, the amount of target mRNA was significantly higher for the strain with the tMOX expression cassette (Calculations in Supplementary Information). The most substantial difference between the two samples was seen 15 min after transcription inhibition, where the tMOX construct was found roughly 900-fold (917 ± 234) more often in the sample than the reference gene, whereas the tCYC1 construct was only present 350-fold (348 ± 26) more often (*p* value = 0.014; Figure 4A). Even after 45 min, there is still a drastic difference in the relative transcript level (tMOX 971 ± 243, tCYC1 547 ± 62; *p* value = 0.04; cp. Figure 4A). As transcript abundance has a major influence on expression levels (Curran et al., 2013), this difference certainly contributes to the elevated GFP expression observed for the tMOX strain compared to the tCYC1 strain (Figure 3). Further, this result implies that the high GFP expression in the tMOX strain is not caused by an increased translation rate but that the regulation occurs at the transcript level. This increased transcript level could either be achieved by an increased transcription rate or through enhanced mRNA stability (Marín-Navarro et al., 2011). Consequently, the degradation of the tMOX and tCYC1 mRNAs over time was assessed by normalizing the amount of target transcript to the reference gene and then to the initial difference in target and reference transcript level before the addition of the antibiotic (Figure 4B). We observed that the tMOX construct degrades more slowly than the tCYC1 mRNA. Comparing differences in the relative transcript decay revealed that at each time point, except 5 min after transcription inhibition, the value for the tMOX construct is higher than the one for the tCYC1 construct (Figure 4B). The most substantial difference between the two constructs was observed 30 min after transcription inhibition (tMOX: 0.66 ± 0.06, tCYC1:0.38 ± 0.04; Figure 4B). Comparing the overall difference between the means for the relative transcript decay of the tMOX and the tCYC1 construct with a paired *t*-test showed that the average values for the tMOX mRNA are significantly higher than for the tCYC1 mRNA (*p* value = 0.015) (calculations in Supplementary Information). Therefore, these results indicate that the two mRNAs with the MOX and CYC1 terminator differ significantly in their stability.

**FIGURE 5:**
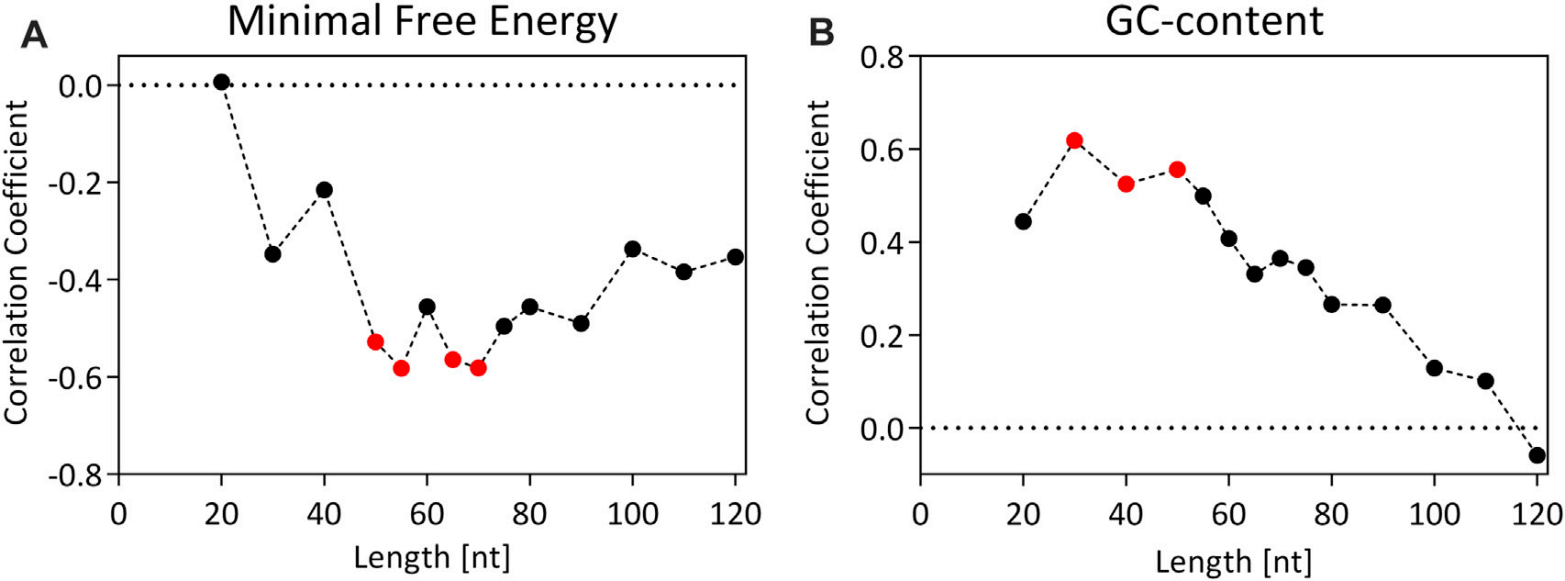
Correlation coefficient (Spearman’s Rho) for the correlation between the GFP signal (RFU) and the minimal free energy (MFE) of the secondary structure **(A)** or GC-content **(B)** for 15 different terminator constructs of fixed lengths. Values of the correlation coefficient can range from −1 to 1. A value of −1 or 1 suggests a perfect negative/positive correlation, while a value of 0 corresponds to no association between the variables. Red dots indicate statistically significant correlations (*p* < 0.05).

However, it is unclear how this decreased transcript decay is achieved. In *S. cerevisiae* it has been shown that strong terminators lead to more stable secondary structures of the 3′UTR, which prevents the rapid degradation of the mRNA through nucleases (Decker and Parker, 1993; Beelman and Parker, 1995). The minimal free energy (MFE) of a sequence can serve as an indicator for the stability of its secondary structure. Therefore, we expected that among the chosen terminators for *O. polymorpha*, stronger terminators would form secondary structures with lower MFEs. The MFEs of the 3′UTR for the terminator sequences in this study were calculated using the RNAfold program (Lorenz et al., 2011). As the MFE is heavily dependent on sequence length, sequences of fixed lengths were extracted downstream of the stop codon of the chosen terminator sequences. 3′UTR lengths in *O. polymorpha* have not been characterized so far. The average reported 3′UTR lengths in the yeasts *S. cerevisiae* and *P. pastoris* range from 30 to 100 nucleotides (nt) (Yamanishi et al., 2013; Ito et al., 2020). Therefore, 3′UTR lengths of 20–120 nt were used to calculate the MFEs. Subsequently, the correlation coefficient Spearman’s Rho was determined between the average GFP-signals and the MFEs, to analyze if there is a correlation between the GFP expression and the MFE of the 3′UTRs (Figure 5A).

There is a maximum correlation between the MFE and the GFP expression strength at a 3′UTR length of 55 nt (Spearman’s Rho = −0.58, *p* value = 0.022), which means that stronger terminators form more stable secondary structures in their respective 3′UTRs. This could be one of the reasons, why an increased transcript abundance and stability was observed for the tMOX.

Similarly, the correlation coefficient between the GC-content of the terminators and the GFP expression level was calculated (Figure 5B). Here, a significant correlation was observed at a 3′UTR length of 30–50 nt, with a maximum correlation at 30 nt (Spearman’s Rho = −0.62, *p* value = 0.016). This could indicate that stronger terminators must have an enhanced GC-content in the first 30–50 nt of the 3′UTR, which is striking as terminator sequences in yeast are generally described as AT-rich sequences (Zaret and Sherman, 1982).

Apart from the secondary structure and GC-content of the 3′UTR, it is known that an intact polyadenylation signal is crucial for efficient transcription termination in yeast. The length of the polyA-tail has been shown to play a key role in determining the half-life of the transcript (Decker and Parker, 1993). However, there is very little information about the polyadenylation signals in *O. polymorpha*. In contrast, polyadenylation signals in *S. cerevisiae* have been described extensively (Guo and Sherman, 1995; Tian and Graber, 2012). Therefore, the chosen terminator sequences in this study were analyzed for elements of the *S. cerevisiae* polyA signals (Supplementary Table S4). This analysis showed that several elements of the *S. cerevisiae* polyA signals could be found in almost all analyzed terminators of *O. polymorpha*. However, these polyA signals are short sequences of 4–10 nt, and thus, their occurrence could also be attributed to chance. Hence, further analysis on the consensus sequence and structure of the *O. polymorpha* polyadenylation signals would be necessary to determine their influence on gene expression.

These findings provide preliminary evidence on how terminators influence gene expression in *O. polymorpha.* There is evidence that the secondary structure and GC-content of the 3′UTR influence the stability of the transcript and hence the expression of a gene. However, further studies will be necessary to determine whether all of the observed differences in gene expression can be attributed to differences in mRNA stability or if more factors, such as polyadenylation signals or binding sites for trans-acting factors, contribute to the observed variations.

### Promoters and Terminators as Independent Genetic Elements

**FIGURE 6:**
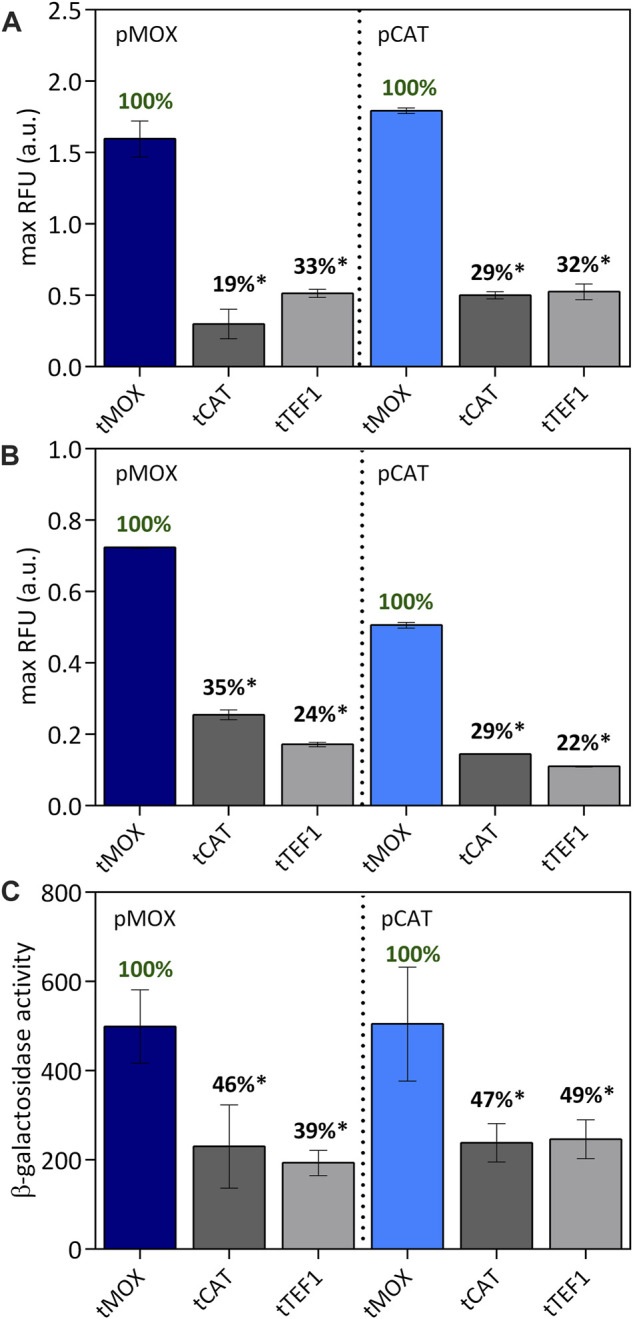
Combination of tMOX, tCAT and tTEF with pMOX and pCAT on methanol **(A)** causing an equally strong induction of the promoters and on glycerol **(B)** causing a medium-strong induction for pMOX and a weak induction for pCAT. The maximum relative fluorescence units are shown here. The strongest terminator was set to 100% for each promoter to allow an easy comparison within one group of promoters. In **(C)** the β-galactosidase activity was determined for the promoter-terminator combinations on methanol as a carbon source. Statistical significance (indicated with *when *p* < 0.05) was tested with an unpaired *t*-test between the max RFU of the various promoters-terminator pairs and the highest max RFU of the dataset (pMOX or pCAT 100% indicated in green) Supplementary Table S8.

Completing our studies, we evaluated different factors for their ability to alter the above-characterized performances of promoters and terminators. For both *P. pastoris* and *S. cerevisiae,* it has been considered that the combination of a promoter with its native terminator could potentially boost gene expression (Curran et al., 2013; Vogl et al., 2016; Ramakrishnan et al., 2020). To identify whether these synergistic effects exist between native promoter and terminator pairs in *O. polymorpha*, two promoters, pMOX and pCAT were selected and coupled with three different terminators, tMOX, tCAT and tTEF1. When cultivated on methanol, both pMOX and pCAT showed the highest GFP expression combined with the terminator tMOX (Figure 6A). For pMOX-tMOX a GFP fluorescence of 1.55 ± 0.17 RFU was observed, which was set to 100% for a better comparison of the terminators (Figure 6A). The combination of pMOX with tCAT and tTEF1 only reached an expression strength of 19% and 33%, respectively (Figure 6A). The same effect was recognized with the pCAT promoter on methanol. The pCAT-tMOX combination generated a GFP expression of 1.74 ± 0.26 RFU and is comparable to the GFP values obtained with the pMOX-tMOX (Figure 6A). The combination of the pCAT with the tCAT and tTEF1 only obtained 29% and 32% of the pCAT-tMOX activity, respectively (Figure 6A). Thus, not the native promoter-terminator combination pCAT-tCAT but pCAT-tMOX generated the highest GFP expression. In a study with *P. pastoris* by Vogl et al. (2016) the AOX terminator outperformed all other terminators when combined with the AOX promoter. The dominant performance of the AOX terminator may be explained by its combination with its native promoter (Vogl et al., 2016). However, no such combinatory effect could be observed in *P. pastoris* when coupling the GAP promoter with its terminator (Ramakrishnan et al., 2020). Likewise, in *S. cerevisiae* studies, no apparent effect could be observed when native and non-native promoter and terminator pairs were combined (Curran et al., 2013). Also, in *O. polymorpha*, we observed no synergistic effect for the CAT promoter when combined with its native terminator.

On methanol, both pMOX and pCAT are exceptional strong promoters and thus do not represent the wide range of expression strength observed in yeast promoters. Therefore, the influence of a terminator might be more profound when paired with a low-expression promoter, which was observed in a study by Curran et al. (2013). To modify the expression strength of our promoters, we evaluated the same set of promoter and terminator pairs on glycerol as a carbon source. Both promoters are strongly induced on methanol; however, when cultivated on glycerol, pMOX is only expressed by 70%, representing a medium-strong expression and pCAT only by 20%, representing a low expression (Figures 1B,D). On glycerol (Figure 6B), the combination of pMOX and pCAT with the tMOX terminator also resulted in the highest expression levels for GFP. Here, pMOX-tMOX generated 0.72 ± 0.00 RFU and pCAT-tMOX 0.51 ± 0.01 RFU. Combined with tCAT and tTEF1, pMOX only generated 35% and 24% of the initial pMOX-tMOX activity, respectively (Figure 6B). For the combination of tCAT and tTEF1, the same trend is recognized: pCAT-tCAT and pCAT-tTEF only obtained 29% and 22% respectively of the activity of pCAT-tMOX on glycerol. Hence, the terminator strength remains unaffected by the promoter located in front of it, even if the promoter strength is altered, for example, through different carbon sources.

The performance of a promoter and a terminator might also be influenced by the gene they control. Therefore, we characterized our promoter-terminator sets not only via the expression of the gene coding for the green fluorescent protein GFP, but also using the lacZ gene coding for the β-galactosidase enzyme. Also with lacZ, the highest expression levels were obtained with the combinations of both the pMOX and the pCAT promoter with the tMOX terminator (Figure 6C). For the pMOX-tMOX combination, a β-galactosidase activity of 499 ± 82.2 was obtained, which is comparable to 505 ± 103 for the pCAT-tMOX construct. Both terminators tCAT and tTEF1 only generated 39%–49% of the β-galactosidase activity reached with tMOX. When comparing the expression of the GFP (Figure 6A) with the expression of the β-galactosidase (Figure 6C), the same trend of the different promoter and terminator sets can be observed: Equally high expression levels are reached with both pMOX-tMOX and pCAT-tMOX while tCAT and tTEF only obtained levels of up to 33% and 49% for GFP and lacZ respectively (Figures 6A,C). We therefore found the same expression pattern for both tested genes coding for GFP and β-galactosidase.

Overall, our findings suggest that promoters and terminators can be applied as independent elements to stir gene expression, not being influenced by one another or by the gene they control.

## Conclusion

Here we provide an in-depth characterization of several promoters and terminators for tuning gene expression in *O. polymorpha*. Both promoters and terminators have been characterized on different carbon sources throughout batch cultivations. This study confirms that the promoters of the methanol utilization pathway in *O. polymorpha* lead to exceptional expression levels during cultivations on methanol. We further found that promoters with a similar expression strength can still vary drastically in the time point of maximal expression. Therefore, our promoter studies underline the powerful influence of the carbon source on *O. polymorpha*’s promoters and the necessity to carefully choose a suitable promoter depending on the application and chosen bioprocess conditions. The power of terminators as independent elements to control gene expression in *O. polymorpha* has been widely underestimated up to now. Our terminator studies show that varying the terminator of an expression cassette can significantly impact gene expression independent of the carbon source. It was shown that stronger terminators stabilize the mRNA and increase the transcript level in the cells, which ultimately leads to a higher gene expression. Thus, terminators provide an additional, more predictable and controllable way of tuning gene expression levels under varying culture conditions. By pairing different promoters and terminators with each other and by expressing two different reporter genes, we could confirm that the promoters and terminators from this toolbox can be applied as independent genetic elements. Hence, one can deliberately mix and match promoter and terminator pairs for gene expression in *O. polymorpha*, simplifying the use of this exciting yeast on our quest toward a sustainable bioeconomy.

## Data Availability

The original contributions presented in the study are included in the article/Supplementary Material, further inquiries can be directed to the corresponding author
