# Supplementary material for: Mix and Match: Promoters and Terminators for Tuning Gene Expression in the Methylotrophic Yeast Ogataea polymorpha
Source: Front Bioeng Biotechnol. 2022 May 10;10:876316. doi: 10.3389/fbioe.2022.876316 (PMC9127203; doi:10.3389/fbioe.2022.876316)
Supplement: Supplementary file 1 [file DataSheet1.docx]

# **Supplementary information**

# **Mix and match: Promoters and terminators for tuning gene expression in the methylotrophic yeast *Ogataea polymorpha***

Katrin Wefelmeier^1^, Birgitta E. Ebert^2^, Lars M. Blank^1*^, Simone Schmitz^1^

*1 iAMB - Institute of Applied Microbiology, ABBt – Aachen Biology and Biotechnology, RWTH Aachen University, Worringerweg 1, D-52074 Aachen, Germany*

*2 Australian Institute for Bioengineering and Nanotechnology, The University of Queensland, Brisbane, QLD 4072, Australia*

*** *Correspondence:* *Lars.Blank@rwth-aachen.de*

This supplementary document contains supporting information for the construction of the integrative plasmids and the confirmation of their correct integration into the genome of *O*. *polymorpha*. All used primer for cloning, colony PCRs or qPCRs are listed in Table S1. Table S2 provides an overview of all used plasmids in this study. Table S3 and Table S4 give information on promoter and terminator sequences, respectively. Furthermore, data on copy number analysis of all constructed strains are presented in Figure S1 & S2. Additionally, this supplementary document provides information on growth rates of *O.* *polymorpha* on different carbon sources (Figure S3), on the substrate consumption (Figure S4&5). Statistical analyses performed in this study are found in Table S5-8.

Table S1: Plasmids

| Plasmid | | Backbone | | Features | | Restriction site  used for linearization | | Origin |
| --- | --- | --- | --- | --- | --- | --- | --- | --- |
| pHIPH4 | | - | | pMOX/tAMO | |  | | (Saraya et al., 2011) |
| pHIPX8 | | - | | pTEF2/tAMO | |  | | see: https://www.rug.nl/research/molecular-cell-biology/research/the-hansenula-polymorpha-expression-system?lang=en |
| pHIPX9 | | - | | pCAT/tAMO | |  | |  |
| pHIPZ7 | | - | | pTEF1/tAMO | |  | |  |
| pHIPZ15 | | - | | pDHAS/tAMO | |  | |  |
| pHIPH18 | | - | | pADH1/tAMO | |  | |  |
| Ubi-GFP | |  | | P_ubiGFP_ADH1ts_ARS1021b | |  | | (Apel et al., 2017) |
| SZ1 | | pHIPH4 | | pMOX_ubiGFP_tAMO | | StuI | | This study |
| SZ2 | | pHIPX8 | | pTEF2_ubiGFP_tAMO | | BclI | | This study |
| SZ3 | | pHIPZ7 | | pTEF1_ubiGFP_tAMO | | BsmI | | This study |
| SZ4 | | pHIPZ7 | | pDHAS_ubiGFP_tAMO | | BsmI | | This study |
| SZ5 | | pHIPZ7 | | pADH1_ubiGFP_tAMO | | AgeI | | This study |
| SZ6 | | pHIPZ7 | | pCAT_ubiGFP_tAMO | | NotI | | This study |
| SZ7 | | pHIPH4 | | pMOX_ubiGFP_tMOX | | StuI | | This study |
| SZ8 | | pHIPH4 | | pMOX_ubiGFP_tTEF1 | | StuI | | This study |
| SZ9 | | pHIPH4 | | pMOX_ubiGFP_tCAT | | StuI | | This study |
| pKW1 | | SZ6 | | pCAT_ubiGFP_tTEF2Sc | | SpeI | | This study |
| pKW3 | | SZ6 | | pCAT_ubiGFP_tTEF1Ag | | SpeI | | This study |
| pKW4 | | SZ6 | | pCAT_ubiGFP_tAOXPp | | SpeI | | This study |
| pKW5 | | SZ6 | | pCAT_ubiGFP_tMOX | | SpeI | | This study |
| pKW6 | SZ6 | | pCAT_ubiGFP_tAMO | | SpeI | | This study | |
| pKW7 | SZ6 | | pCAT_ubiGFP_tCYC1 | | SpeI | | This study | |
| pKW8 | SZ6 | | pCAT_ubiGFP_tTDH3 | | SpeI | | This study | |
| pKW9 | SZ6 | | pCAT_ubiGFP_tRPS2A | | SpeI | | This study | |
| pKW10 | SZ6 | | pCAT_ubiGFP_tRPS25A | | SphI | | This study | |
| pKW11 | SZ6 | | pCAT_ubiGFP_tRPS3 | | SpeI | | This study | |
| pKW12 | SZ6 | | pCAT_ubiGFP_tPMA1 | | SphI | | This study | |
| pKW13 | SZ6 | | pCAT_ubiGFP_tPGK1 | | SpeI | | This study | |
| pKW16 | SZ6 | | pCAT_ubiGFP_tFMD | | SphI | | This study | |
| pKW17 | SZ6 | | pCAT_ubiGFP_tCAT | | SphI | | This study | |
| pKW18 | SZ6 | | pCAT_ubiGFP_tTEF1 | | SphI | | This study | |
| SZ10 | SZ7 | | pMOX_lacZ_tMOX | | SphI | | This study | |
| SZ11 | SZ8 | | pMOX_lacZ_tTEF | | SphI | | This study | |
| SZ12 | SZ9 | | pMOX_lacZ_tCAT | | SphI | | This study | |
| pKW23 | pKW5 | | pCAT_lacZ_tMOX | | SphI | | This study | |
| pKW24 | pKW17 | | pCAT_lacZ_tCAT | | SphI | | This study | |
| pKW25 | pKW18 | | pCAT_lacZ_tTEF | | SphI | | This study | |

Table S2: Primers

| Name | Sequence | Purpose |
| --- | --- | --- |
| SZ121_pHIPH4_fwd | ATACAAATAGAAGCTTGCATGCCTGCAG | Gibson Primer |
| SZ122_pHIPH4_rev | AAATCTGCATGTTTTTGTACTTTAGATTGATGTCACCAC | Gibson Primer |
| SZ123_pHIPX8_fwd | ATACAAATAGGGATCCAAGCTTGCATGC | Gibson Primer |
| SZ124_pHIPX8_rev | AAATCTGCATTTTGTAGTTTTCAGTCTTTGAAAAAGC | Gibson Primer |
| SZ125_pHIPZ7_fwd | ATACAAATAGGATCCAAGCTTGCATGCC | Gibson Primer |
| SZ126_pHIPZ7_rev | AAATCTGCATCTACGAATGTACTAATTAAAAGATCAGAAAAAATC | Gibson Primer |
| SZ127_pHIP18_fwd | ATACAAATAGGTCGACTCTAGAGGATCG | Gibson Primer |
| SZ128_pHIP18_rev | AAATCTGCATTTTTAAATTGATTGATTGATTGATAGAAG | Gibson Primer |
| SZ129_UbiGFP_H4_fwd | GTACAAAAACATGCAGATTTTCGTCAAG | Gibson Primer |
| SZ130_UbiGFP_H4_rev | ATGCAAGCTTCTATTTGTATAGTTCATCCATGC | Gibson Primer |
| SZ131_UbiGFP_X8_fwd | AAACTACAAAATGCAGATTTTCGTCAAG | Gibson Primer |
| SZ132_UbiGFP_X8_rev | GCTTGGATCCCTATTTGTATAGTTCATCCATGC | Gibson Primer |
| SZ133_UbiGFP_Z7_fwd | ACATTCGTAGATGCAGATTTTCGTCAAG | Gibson Primer |
| SZ134_UbiGFP_Z7_rev | AGCTTGGATCCTATTTGTATAGTTCATCCATGC | Gibson Primer |
| SZ142_pHIP18_fwd | ATACAAATAGCCCACACACCATAGCTTC | Gibson Primer |
| SZ143_pHIP18_rev | AAATCTGCATTTTTAAATTGATTGATTGATTGATAGAAGG | Gibson Primer |
| SZ144_UbiGFP_18_fwd | CAATTTAAAAATGCAGATTTTCGTCAAG | Gibson Primer |
| SZ145_UbiGFP_18_rev | GGTGTGTGGGCTATTTGTATAGTTCATCCATGC | Gibson Primer |
| SZ176_DHAS_fwd | GCCGCTCTAGGCGGCCGCTCGAGAGCCG | Gibson Primer |
| SZ177_DHAS_rev | AAATCTGCATGGGAAGAAAAGACAGAGATGACTGTGTAGCAGACG | Gibson Primer |
| SZ178_pHIPZ7_uGFP_DHAS_fwd | TTTTCTTCCCATGCAGATTTTCGTCAAGACTTTGACCGG | Gibson Primer |
| SZ179_pHIPZ7_uGFP_DHAS_rev | GAGCGGCCGCCTAGAGCGGCCGCCACCG | Gibson Primer |
| SZ180_CAT_fwd | GCCGCTCTAGAAGCTTATCGCCTCGACG | Gibson Primer |
| SZ181_CAT_rev | AAATCTGCATCTTTGTTCAACAGTGATTAGCC | Gibson Primer |
| SZ182_pHIPZ7_uGFP_CAT_fwd | TTGAACAAAGATGCAGATTTTCGTCAAGACTTTGACCGG | Gibson Primer |
| SZ183_pHIPZ7_uGFP_CAT_rev | CGATAAGCTTCTAGAGCGGCCGCCACCG | Gibson Primer |
| SZM44_pHIPH4_tMOX_I_FOR | GCATGGATGAACTATACAAATAGGGAGACGTGGAAGGACATACCG | Gibson Primer |
| SZM45_pHIPH4_tMOX_I_Rev | CTCCTGCGCAGGAGGGACAACGTTCTTAGAAGCGGCC | Gibson Primer |
| SZM46_pHIPH4_tTEF1_I_FOR | GCATGGATGAACTATACAAATAGACCACTGCTATTCAAAACAATAGACCATG | Gibson Primer |
| SZM47_pHIPH4_tTEF1_I_Rev | CTCCTGCGCAGGAGGGGAAAATTTGAAGGTGTCACGTTGT | Gibson Primer |
| SZM48_pHIPH4_BB_FOR | CCTCCTGCGCAGGAGC | Gibson Primer |
| SZM49_pHIPH4_BB_Rev | CTATTTGTATAGTTCATCCATGCCATGTGTAAT | Gibson Primer |
| SZM50_pHIPH4_tCAT_I_FOR | GCATGGATGAACTATACAAATAGTCATCGATAAGTATTTATTGAATCAGTGTTCGTATACTATATTTTTT | Gibson Primer |
| SZM51_pHIPH4_tCAT_I_Rev | CTCCTGCGCAGGAGGCGCTGGACGGTGACGG | Gibson Primer |
| SZ146_UbiGFP_rev | TACCACCTCTTAGCCTTAGC | Colony PCR Primer |
| SZ52_TEF1-gen-for | CGACGTGACCAATAACAAC | Colony PCR Primer |
| SZ53_TEF1-gen-rev | GGGCATCATGTTTAGTCTTTG | Colony PCR Primer |
| SZ57_TEF2-gen-for | TGATATCTTTGGCATCATTCG | Colony PCR Primer |
| SZ58_TEF2-gen-rev | CCGGTAGTGGTAGATTTACCAG | Colony PCR Primer |
| SZ55_AOX-gen-rev | ACCTCCACCAACAACAATG | Colony PCR Primer |
| SZ56_AOX-gen-for | CGCCAAGGGATACAAGTAG | Colony PCR Primer |
| SZ111_DHAS-gen-rev | CGGCCTATTGTCATCAGTG | Colony PCR Primer |
| SZ112_DHAS-gen-rev | TGCAGAGTATGGTTCCTAGAG | Colony PCR Primer |
| SZ194_Cat-gen-for | ACTCCGTCGAACGTGATG | Colony PCR Primer |
| SZ195_Cat-gen-rev | ATCCGTTGGGTGGTGAAG | Colony PCR Primer |
| KW001_pHIPZ9_uGFP_fw | CCCCACACACCATAGCTTCAAAATG | Gibson Primer |
| KW002_pHIPZ9_uGFP_rv | CTATTTGTATAGTTCATCCATGCCATGTGTAATCC | Gibson Primer |
| KW003_tTEF2Sc_fw | GCATGGATGAACTATACAAATAGGAGTAATAATTATTGCTTCCATATAATATTTTTATATACCTCTTATTT | Gibson Primer |
| KW004_tTEF2Sc_rv | GCTATGGTGTGTGGGGGATGAGGCCGTCTTTTGTTGATAGC | Gibson Primer |
| KW007_tTEF1Ag_fw | GCATGGATGAACTATACAAATAGATCAGTACTGACAATAAAAAGATTCTTGTTTTCAAGAAC | Gibson Primer |
| KW008_tTEF1Ag_rv | GCTATGGTGTGTGGGGCGTTTTCGACACTGGATGGCG | Gibson Primer |
| KW009_tAOXPp_fw | GCATGGATGAACTATACAAATAGTCAAGAGGATGTCAGAATGCCATTTGC | Gibson Primer |
| KW010_tAOXPp_rv | GCTATGGTGTGTGGGGTCTCACTTAATCTTCTGTACTCTGAAGAGGAGTG | Gibson Primer |
| KW011_tMOX_fw | GCATGGATGAACTATACAAATAGGGAGACGTGGAAGGACATACCG | Gibson Primer |
| KW012_tMOX_rv | GCTATGGTGTGTGGGGGACAACGTTCTTAGAAGCGGCC | Gibson Primer |
| KW013_tAMO_fw | GCATGGATGAACTATACAAATAGCCCGGGCCTGGACAT | Gibson Primer |
| KW014_tAMO_rv | GCTATGGTGTGTGGGGGATCTGAACCTCGACTTTCTGGATCAG | Gibson Primer |
| KW015_tCYC1_fw | GCATGGATGAACTATACAAATAGTTCATATGATCGATGTAATAAATTATAATAGAGGTGCTCTGAAGG | Gibson Primer |
| KW016_tCYC1_rv | GCTATGGTGTGTGGGGCAACAGCTGGAGGAAAAACACGT | Gibson Primer |
| KW017_tTDH3_fw | GCATGGATGAACTATACAAATAGGCTCGGCTCCATCTACATATTTACG | Gibson Primer |
| KW018_tTDH3_rv | GCTATGGTGTGTGGGGGCTCCGTAACACGTTACGGAC | Gibson Primer |
| KW019_tRPS2A_fw | GCATGGATGAACTATACAAATAGGTATGGGTAATATCTCTGTTTAGATGGAGCTG | Gibson Primer |
| KW020_tRPS2A_rv | GCTATGGTGTGTGGGGGCGTAAGAATATTTTTCGATGGATTCCATCA | Gibson Primer |
| KW021_tRPS25A_fw | GCATGGATGAACTATACAAATAGACAGACTAGTATAGTATAATACATGATGCAGAAGTGT | Gibson Primer |
| KW022_tRPS25A_rv | GCTATGGTGTGTGGGGTTCGCCAACGAAGTGTTCTGGG | Gibson Primer |
| KW023_tRPS3_fw | GCATGGATGAACTATACAAATAGGTGTATAGCGCATAATGAACATATAAGTCTATTAGTCTT | Gibson Primer |
| KW024_tRPS3_rv | GCTATGGTGTGTGGGGACACCTTTACAAGATGTTCCATGGC | Gibson Primer |
| KW025_tPMA1_fw | GCATGGATGAACTATACAAATAGGCCAGCACTTGAATGGGTTAATAGAG | Gibson Primer |
| KW026_tPMA1_rv | GCTATGGTGTGTGGGGGTTGATCTGAAACGCGACTTCACC | Gibson Primer |
| KW027_tPGK1_fw | GCATGGATGAACTATACAAATAGACGTGAAATCTATATACTGAGAAAAAAAATAATTAATTTTAATATAAAC | Gibson Primer |
| KW028_tPGK1_rv | GCTATGGTGTGTGGGGCAATGTTGGATTTCGTGCGTGTT | Gibson Primer |
| KW029_GFP_fw | CTTCTTGAGTTTGTAACAGC | Colony PCR Primer |
| KW042_tFMDHp_fw | GCATGGATGAACTATACAAATAGGCGGTCTTGGAGGAGCT | Gibson Primer |
| KW043_tFMDHp_rv | GCTATGGTGTGTGGGGTATCTCGAGCCCCACGGCG | Gibson Primer |
| KW044_tCAT_fw | GCATGGATGAACTATACAAATAGTCATCGATAAGTATTTATTGAATCAGTGTTCGTATACTATATTTTTT | Gibson Primer |
| KW045_tCAT_rv | GCTATGGTGTGTGGGGCGCTGGACGGTGACGG | Gibson Primer |
| KW046_tTEF1_fw | GCATGGATGAACTATACAAATAGACCACTGCTATTCAAAACAATAGACCATG | Gibson Primer |
| KW047_tTEF1_rv | GCTATGGTGTGTGGGGGGAAAATTTGAAGGTGTCACGTTGT | Gibson Primer |
| KW050_GFP2_fw | GTGAAGGTGATGCAACATACGG | qPCR Primer |
| KW051_GFP2_rv | ACAAGTGTTGGCCATGGAAC | qPCR Primer |
| KW060_TAF10_2_fw | CTCCCATTTTGCCCCAATTC | qPCR Primer |
| KW061_TAF10_2_rv | ATGATCGGTGTGAACTCCTC | qPCR Primer |
| KW062_UBC6_1_fw | TGGGCAGTATCATGGCTTAG | qPCR Primer |
| KW063_UBC6_1_rv | GGGTGTTCACTTGAAACCTG | qPCR Primer |
| SZ50_pEM7-rev | ACTATGCCGATGATTAATTG | Colony PCR Primer |
| SZ291_H4_LacZ_tMOX_bb_for | TGGTCTGGTGTCAAAAATAAGGAGACGTGGAAGGACATACCG | Gibson Primer |
| SZ292_H4_LacZ_tMOX,CAT,TEF_bb_rev | GAATCCGTAATCATGGTCATGTTTTTGTACTTTAGATTGATGTCACCACCG | Gibson Primer |
| SZ293_H4_LacZ_tCAT_bb_for | TGGTCTGGTGTCAAAAATAATCATCGATAAGTATTTATTGAATCAGTGTTCGTATACTATATTTTTT | Gibson Primer |
| SZ294_H4_LacZ_tTEF1_bb_for | TGGTCTGGTGTCAAAAATAAACCACTGCTATTCAAAACAATAGACCAT | Gibson Primer |
| SZ295_pHIPH4tMOX-for | GGAGACGTGGAAGGACATACCG | Gibson Primer |
| SZ296_pHIPH4tMOX-rev | GTTTTTGTACTTTAGATTGATGTCACCACC | Gibson Primer |
| KW144_pKW17_tCAT_bkb_fwd | TGGTCTGGTGTCAAAAATAATCATCGATAAGTATTTATTGAATCAG | Gibson Primer |
| KW145_pKWxy_bkb_rev | GAATCCGTAATCATGGTCATCTTTGTTCAACAGTGATTAG | Gibson Primer |
| KW146_lacZ_rev | TTATTTTTGACACCAGACC | Gibson Primer |
| KW147_pKW5_tMOX_bkb_fw | TGGTCTGGTGTCAAAAATAAGGAGACGTGGAAGGACATAC | Gibson Primer |
| KW148_pKW18_tTEF1_bkb_fw | TGGTCTGGTGTCAAAAATAAACCACTGCTATTCAAAAC | Gibson Primer |
| KW103_pTEF1_rv | TGAAGCTATGGTGTGTGG | Colony PCR Primer |
| KW153_lacZ_fw | ATGAACGGTCTGGTCTTTGC | Colony PCR Primer |
| KW062_UBC6_1_fw | TGGGCAGTATCATGGCTTAG | qPCR Primer |
| KW063_UBC6_1_rv | GGGTGTTCACTTGAAACCTG | qPCR Primer |
| KW151_lacZ2_qPCR_fw | TTTCATCTGTGGTGCAACGG | qPCR Primer |
| KW152_lacZ2_qPCR_rv | TTCTCCGGCGCGTAAAAATG | qPCR Primer |

Table S3: Promoter sequences

| *Genetic Part* | *Description* | *Sequence* |
| --- | --- | --- |
| pTEF2 | Promoter of the translation elongation factor 2 from *O. polymorpha* (Hanpo2_94438) | GGCCGCTCTAGAGCTTGCACGCATACGTTCTCCGTACCAAGACTTGATTGTCTCCCTCAAAGTAAAAATTAAGGTAAGACTCGTTGGCTCCTATTAAATTCATTTGAATAAAGATATCATTTGTCTTGCATCATAAAGCGTGCCTTCAATACCTAGAGAATTTAGATTTACTTAACTCTGAAAATGATCAGGCCCCGAAAACCAGTCATTTTACTTGCTTCTGTGGTCTGTATTCTTGTGGTTTTTGGAATTGTAAGCTCTCGAAATGAGAAATTAAAAGCAGAGATACAAGAGGAGGCAAACCATTTGCTTTATCAATGGGAAACTCTCGAAAAGACTTCCAAGTTTGTTCACAAGCAAAACTATACAAGTGAAATTTCACCCGAACCTTGTACTGTGATAAATCCCCTGACCAATCAATTTTTTGATCTTCGACCTTTAGGTGCATTGGGAAACGATGGGCTGGTACAGGCATGGAATGCACGCGGCTACGATTATGGTCGAAACTTTTCCATAGGTATTTGTTCGACCCCATTAAAACAGCCTCAAAGTCTTCCAGAGTCAGATTTTGAAGGTGTCACAAATAAAAGTGAGGTTGGTGGTTATTATACTGATACAACAGGTCACAAAAAATCCATAGGACAGATATCAACGACTCCAAAATTCAGAGGACGGAAGCTGGTTCTTGAATACACAGACGGTAGTGTTTGTGAAGGCTTCAAAAATGATGGCTCACTCAAAAAGTCGACCATTTTATCGTTTGTTTGTGATCGGGAGATTATGACAAAAGCATCGATCTCTTACGTGGGTTCCCTACATGATTGTTCCTACTTTTTTGAGGTGAGAACTATTCATGGGTGTCCGACAGCCGCAAAGAAAGACGACAAGGCAATAATCTGGATTTTCCTTTTCATTTGCTTGTGTGCAGTTGCGGTGTTCTTTGGGGGAGGTTTTATTTATTCACTTTTCAAATATAACCAGGGCCGTCAATCAGTTTCTCACTGGAAACGCAATTCTTTCACAGCTGGAGTGAAAAACCTTGCTCAGGTAAGTCAACGTGAGGAGATTTACAAATAACTAGATACTAATATCGACTCAATCGGCTATAGAGGGAATTAGTGCACAATCAATATCGTGATAATGTGCCAGAATTCAACAACTTTTTCAGCTCATTTTCAGGAGATGAAAACGAGTTGATTGATGCACTGGATGCGCAACAGAATGAAGACTAATAGACCATTTAGCAGTCTTTATCGAAGGTGAACAATTAAGTTAAATCTAATTACCCGGCATTAACTTCCGGGTAACCAAACTTTAAGGCCGAAAATTTTTGCGATGAGGCGAATTGAATAAGGTGGGAATTGACCTGAAAAATTTTCTTCAGCAGCTTTTTCAAAGACTGAAAACTACAA |
| pTEF1 | Promoter of translation elongation factor 1 from *Ogataea polymorpha* (Hanpo2_8811) | CATGGAACCAAGACCCATGACGTTGTTTCTTGATGATCTCTTTTCGTTTCTGCTTTAATGTTTTTATTTGCTGATCGATTGCCTCTACATCGTTTTTCTGCACATCGCTTGAATCGCAGTCGTTATTTGTTTCCACTTTTTCTTTCGGGTTTGACAACCTCCTCGATTCGTTGACAGTATCAAACACATCGGTAAAAGAACTGCTACGTGAAGTCTTGTCAGGCTCAGGTATCATTTCCTCTGACTTTTCTTCCTCCTTTTGGCTTTCATGAGAGAACTGCTTCTCGGAATCACACACACTATCTAGTGTTCGGTCTGCATCTTCTGGCGTCGAATGCTCGGGCTCAAAGCATAGAATGTCCAAGTTTGAGGGCTCTGTTTGGAACTGCTCCAATTCTTTGCTCGTTGGTCTTTGGAATAAATTGTTGTTGAGTAGTTTTGATCGAACTGGAGAGTCCCTATAATTGCGATTTGTATGTGCTGCAAGAATATGTGTAGAAGCAGTATTATGGATACTTCTCAAAGAGCTACTTCTCACCGATACCTCGTCCTTGAACGAGACCGTTTTTTTACCCAATTGCTCCTCGTCCACTAGTTTGTTTGGTTTCATGATGGACTTCAGCATTGAGCTATGTAGATCACCTTCTGGGCCTCCTGGGTTGTCTGGCTCAACTTGCTCTTTGTCATCATGTACAAAGGATAATGGGATGGTGGTGACTTTTGGAAACTGTACAATGGGGTGAGTATTGAGTTGTAATGTCCCCCAGACATTCAGAAGGTCAGGCTTGGGAGCAGGCTTTTTACTCAGCCTCAAACTACCGTTTCTCCGCCTTCGTCTTCTTGACACAGTTGATACGCTGTTGGAAATGGAATGGTCGTCAGTCTCGTTTAGCCCTAAAGACACCGTTGCCATAATTAGGGATAAATCTATTTAGGCTCCTTATTTTTCACTCGGCGCGAAAAAAAATGGGCACACGGCGATCGACGAGAGGTCGATCGTCTCTACGTTCCAGCTACATGGCAGTTCTAAGACGGGAAGTAAGATGACACTAGTAGATGTTTTGCAAATTAGGATCACAGGCCTGCTCCAGAAAACTTTTTATCCTTCAGCGGAAGGTCTGTCCAGGGGCACAAATCTCAAACAGAGCAAAGGCACCCTTACCCTCTTATCTCCTTGATGAATTTTTTTTTTCTTTTAAATTCTTTTTTAGAAGGCCGGGTAACAACTTTAAGAACCTGTTTCAGCTGTCCCCAGGAAGCTCCATTGGCAGTTCCAGTACGTTGTAGATGTGTATAGTGTGCTGAAAAGCGTTGTCCCTGGTTTTTTCAAGCAAAATCTTCGTCTCGGAGCTGGATAGCCCACCAAGGTATTGTTCCTGTGCGTAATTTTTGGCACGCAGACGACTCGAATAAGTTTGGCAATAAAAAAATTTTTTTCACTATATAAAGAGGAGACATTCCCACATGAGATTTTTTCTGATCTTTTAATTAGTACATTCGTA |
| pMOX | Promoter of the methanol oxidase from *O. polymorpha* (Hanpo2_76277) | TCGACGCGGAGAACGATCTCCTCGAGCTGCTCGCGGATCAGCTTGTGGCCCGGTAATGGAACCAGGCCGACGCGACGCTCCTTGCGGACCACGGTGGCTGGCGAGCCCAGTTTGTGAACGAGGTCGTTTAGAACGTCCTGCGCAAAGTCCAGTGTCAGATGAATGTCCTCCTCGGACCAATTCAGCATGTTCTCGAGCAGCCATCTGTCTTTGGAGTAGAAGCGTAATCTCTGCTCCTCGTTACTGTACCGGAAGAGGTAGTTTGCCTCGCCGCCCATAATGAACAGGTTCTCTTTCTGGTGGCCTGTGAGCAGCGGGGACGTCTGGACGGCGTCGATGAGGCCCTTGAGGCGCTCGTAGTACTTGTTCCGTCGCTGTAGCCGGCCGCGGTGACGATACCCACATAGAGGTCCTTGGCCATTAGTTTGATGAGGTGGGGCAGGATGGGCGACTCGGCATCGAAATTTTTGCCGTCGTCGTACAGTGTGATGTCACCATCGAATGTAATGAGCTGCAGCTTGCGATCTCGGATGGTTTTGGAATGGAAGAACCGCGACATCTCCAACAGCTGGGCCGTGTTGAGAATGAGCCGGACGTCGTTGAACGAGGGGGCCACAAGCCGGCGTTTGCTGATGGCGCGGCGCTCGTCCTCGATGTAGAAGGCCTTTTCCAGAGGCAGTCTCGTGAAGAAGCTGCCAACGCTCGGAACCAGCTGCACGAGCCGAGACAATTCGGGGGTGCCGGCTTTGGTCATTTCAATGTTGTCGTCGATGAGGAGTTCGAGGTCGTGGAAGATTTCCGCGTAGCGGCGTTTTGCCTCAGAGTTTACCATGAGGTCGTCCACTGCAGAGATGCCGTTGCTCTTCACCGCGTACAGGACGAACGGCGTGGCCAGCAGGCCCTTGATCCATTCTATGAGGCCATCTCGACGGTGTTCCTTGAGTGCGTACTCCACTCTGTAGCGACTGGACATCTCGAGACTGGGCTTGCTGTGCTGGATGCACCAATTAATTGTTGCCGCATGCATCCTTGCACCGCAAGTTTTTAAAACCCACTCGCTTTAGCCGTCGCGTAAAACTTGTGAATCTGGCAACTGAGGGGGTTCTGCAGCCGCAACCGAACTTTTCGCTTCGAGGACGCAGCTGGATGGTGTCATGTGAGGCTCTGTTTGCTGGCGTAGCCTACAACGTGACCTTGCCTAACCGGACGGCGCTACCCACTGCTGTCTGTGCCTGCTACCAGAAAATCACCAGAGCAGCAGAGGGCCGATGTGGCAACTGGTGGGGTGTCGGACAGGCTGTTTCTCCACAGTGCAAATGCGGGTGAACCGGCCAGAAAGTAAATTCTTATGCTACCGTGCAGTGACTCCGACATCCCCAGTTTTTGCCCTACTTGATCACAGATGGGGTCAGCGCTGCCGCTAAGTGTACCCAACCGTCCCCACACGGTCCATCTATAAATACTGCTGCCAGTGCACGGTGGTGACATCAATCTAAAGTACAAAAAC |
| pADH1 | Promoter of the alcohol dehydrogenase 1 from *O. polymorpha* (Hanpo2_82460) | GGCCGCCCCCTGCATTATTAATCACCACCCCGTCTACGATGACAGGCTCGCGACTGCAGAGATGGCCTTTGTTACGGGCAATACATTTGTCACACGGCACCTCGAAGTTGCACTTAACTTTGCGCTTCTTGCAGCTGAGACATGTCTTGGAAATACGAGTAGTACGCCGTAGCTTGGTTATGTCGAGCCTAGTAAACTCCGCAGGAGAATTCCTCTTCTCCGAAGGCACTCTCTCCGCAGAAGCCATAAATTATTTGAAATCAGAAATGTGAGCGATATAAACACCCCTGCACGGGGCTGCACCCCGATTTTTTTCGGGTGATCGTATGATGCTACTATGAGCCCGCAGATAACATCTGACTTTACATTAAGGCAAAATTCTGGTGTAGGGTGTCCGCAGGCCGAGTAATGCTGACCGGTACGCACCTTTTTCGTCCGTTGTGGTGTGCTGCTTCATGGTCAATTTTTTTTAGCTCAGATGACATCGGAAGGTGTTCCTACAAAGCACACCTCCAATTCGCCGTAAAACCTCGGAGTAAATCATTCTCCCTGTAGTGTAAACTAAGCTGATGAGGCGTTCGGCAGATCCAATTGGACACACCCCGTCATGGGGTGTGAAATACCCCGCCACGGCCATCCGGCAGCCCCACTCCACCACTTTGGGTTGAATATGGTCCAATTGTTATGTGCAAATTTTCGGACATTGGAAATCACCCACTCGGAGTAAAGCAACTCGTAGTGACTGAAAAATAAGACGTCATCATTAATTAGGCTGCAACAGACATATAAATACGAGACACTTTTCCCTCTTCTTAGGCATCCTTCTATCAATCAATCAATCAATTTAAAA |
| pCAT | Promoter of the catalase of *O. polymorpha* (Hanpo2_95533) | AAGCTTATCGCCTCGACGGTGTAAACAGCCTGCAGCTCGCTGGGCTTGAGCGTCGGGTCGGGCTGGAGCGAGCTCTGTGCAATGCGGGCCTGTTTTGCCGAGCAAAATGGGATCCAGTACGATCTAGACGGTCAGACAACAACAATGACGACTACTTACAGTTTGTGAGAGAACACCATAAGAAAAAGATTGTTGCACAGCCCGCTTTGAGAAGGAAATAACCGTAGCCTATATCAGTCACTCCTCGCACCGCAACGATTCTGAAATGATTGCGATTTCCTAATCCAGCCTTACAAATTCAAGCAACCCAATTGGCCCCACAAACGCAGCAAGTCCAAAAAATGTGCTCGACAAGTTTGTGGATTTTGCTGTACCGCGGCGGGTGAACGATGCAAAGTGCTGTTTCTTGAAGACAATTGGTCATCAGCATTCAGCATTCAGCATGGATCTCGTGATCGGAGAGCAACTGTAGAGTGACTCTTATTAATTATGCAACCAAGTCCAGAAACTCTCCTCAGATATGTCAGCAATTTTGAAACTAGTAAAATAATAGTCTATGCATGCAGGCGGGGGCAACAGTACTAATTGAAGCCTAAGCAAAAATATATAAAGGCTCCCGGCCGCACCCCAACTTGGCTAATCACTGTTGAACAAAG |
| pDHAS | Promoter of the dihydroxyacetone synthase from *O. polymorpha* (Hanpo2_95557) | GCGGCCGCTCGAGAGCCGTGGAACAGAGACCCCTGAATCTCTTGCCAAGCGGCTTGCTGCTGCATCTGCGGAGATGGAGTACGCCAGGGCAGTGGACACGACAAGGTCATTGTCAACGATGACCTTGAGAAGGCGTACTCTGAGCTGAAGGAGTTCATTTTCGCCGAGCCCATCTAAGCATTCATAAATTTTTAATATCTAGAGCTCTCATACGGGACAGTATCTCCTCCAACCTTGCGTCAAGCTTGTCCTCTTCATGCTCCTCAACAGTCATGGCATCCAGCTGCTGCTGCTTTTGCTCCAGCCTGGCATATATGTCGCCATACAGCTTGAGTTGGATTTTGATGAAACTCTCAAAGGTAGGGTCCACCAGTGACAGTCGCAGCGCAATGAACTGCTCGATTTCGTTCTTGAGCCGTGTGTTGATGTCCGTGTAGATATTTTCTGCCTCGTCGTACTCAACTTTGAACTTCTGCAGCTTGTCCAGGCTCTTCTGTAACTGGTCTGTTTTCTCGGTGTGATGCTGCTCGGTCACCTGTCGCTCAATCGCTTCGTACTCGCTCTGCAGCTTCGAAACCTTGAATCGTGAAACGTCGTAATCCACCTTTTTGCGTGCGCGCTTCTTGATCAGCTTGTTGATCTCGTCGTTGTACTTCTTCAGCTCGTTAATCGGCTCCACGACCGTGATGCTCATTGGCTCCAGAATTTCTGGCAGAATATTGTCTTTGATGTCTTCCACCATCTGCAGATAATTCAGAGAAATACCATCTCTGGGGTTCACCTTGTGCTCTTCTGGCCGTTCCGCAGCTTCCGACCGCTTATCAGCCTTGAGCTCAAAGCTATAGTCTCCGTAAAACGAGTCCAGTGTTCTAGCCATATTTATCTGAGTCTCGAGCAGATTCTCCGAAATTGCCCACAAAACGGCCTAGTTCCTGGTCCAGCTCGTTGGTGTAAGTCTCGAGTTTGCGGAAATTGGCCTCCTGGACGTCAAACTCAGGATCAACAGAGGGCTCACCTTTGTTTGTGCGTAGTATCACATGTGCTCCGGCACGATTGACAGCTTTTTTAAACCCAACCCATGACATGTCGAGGAAAGGGTCGTTTCGGGGAGTTAAATATTTTTGGCTATGTAGCAGACATGTTTCGACGCTGGCGTCGCGTCGATCGGAAAATATTACCCCAGGAACAAGCACTTGCTTGGGTTAGCCACCACCCTGCGCAAGCCTTTTTGCCGGCTCTACACAGGGCCAATGAAATCTGGGCGGAATCTGAAACCGATGAAACGGACGACACTGGCAACAAGCTCACTGCACTATTTTTTTTTTCTAGTGAAATAGCCTATCCTCGTCTCGCTCCCCTCATACCTGTAAAGGGGTGCAATTTAGCCTCGTTCCAGCCATTCACGGGCCACTCAACAACACGTCGGCTACCATGGGGTGCTTGGGCACCAAAAGGCCTATAAATAGGCCCCCATCCGTCTGCTACACAGTCATCTCTGTCTTTTCTTCCC |

Table S4: Terminator sequences including analysis for S. cerevisiae polyA signals

| *Genetic Part* | *Description* | *Sequence* |
| --- | --- | --- |
| tMOX | Terminator of methanol oxidase from *Ogataea polymorpha* (Hanpo2_76277) | GGAGACGTGGAAGGACATACCGCTTTTGAGAAGCGTGTTTGAAAATAGTTCTTTTTCTGGTTTATATCGTTTATGAAGTGATGAGATGAAAAGCTGAAATAGCGAGTATAGGAAAATTTAATGAAAATTAAATTAAATATTTTCTTAGGCTATTAGTCACCTTCAAAATGCCGGCCGCTTCTAAGAACGTTGTC |
| tAMO | Terminator of peroxisomal primary amine oxidase from *Ogataea polymorpha* (Hanpo2_86266) | CCCGGGCCTGGACATCCAGCCTTCCTACGCCATGACCACCTCCGAGGCTAAGAGGGCCGTGCACAAGGAGACCAAGGA**CAAA**ACCTCGAGACTTGCCTTTGAAGGCTCTTGTTGCGGTAAATAAG**TATATA**GGACACGACAATCTAGTAATCTCCACTATTGACGAGCTCGTCGAACTGCGAAAATAGGTTTTCCATCTGGTCTGTAGGCATCAGCCCGGCGTCATCCTCCTGCGCAGGAGCAGCGGGCTCAGGGCCGGCCTGGGCGGGCTGATCCAGAAAGTCGAGGTTCAGATC |
| tCYC1 | Terminator putative mitochondrial cytochrome c from *Ogataea polymorpha* (Hanpo2_83364) | TTCATATGATCGATGT**AATAAA**TTATAATAGAGGTGCTCTGAAGGTTGCTTAGACATATGTAGCCAATAGTAGTCGGATGAATACGTCTTAGATGCCTGCCGTCGATCGACGCGTCGCAACAGTGTAAATCCGCACAGAGCAGTCGCCGCTTCCCGCCGCCAGCAGCAGCTTGTCCGAATTCTCGGGCTTTGGACACCAGTTCAGCCGCGCGACCTTGTCGTCCGGTACGTGTTTTTCCTCCAGCTGTTG |
| tTDH3 | Terminator of glyceraldehyde-3-phosphate dehydrogenase  from *Ogataea polymorpha* (Hanpo2_89929) | GCTCGGCTCCATCTACATATTTACGGCTTAACTGATTTTTATAGTTAAGGAGAAAAAAAAGCTCAACATACGTCATTATTATCGTACGCTCTTTGGTGTTTCGAGCCTGGCTGCCATGTTCAAAAACACTATCGTTGCTTGCTATGTAAAAATTATTTGATTAACCTAGCACTTGATTCCTTTATAATCTTTGTGGTACCTCGTGGCAGGGACGTGGTCTGGAATTATAGTCCGTAACGTGTTACGGAGC |
| tRPS2A | Terminator of putative protein component 2A of the 40S ribosome subunit from *Ogataea polymorpha* (Hanpo2_17063) | GTATGGGTAATATCTCTGTTTAGATGGAGCTGTAGAAGGTGGCCGCTTTGC**AAAAAA**CCAACTCTGCGGCTCGTTGTCTGCCTTTAATTCAGTGATTTCCTGAA**AATAAA**ATGA**AATAAA**ATAAAATT**AAAAAA**ATTATCC**TATATA**CTTGATTGAATCGAAAAAGTATAACGGCCATGCTTTCTACCTGTGTGACAGCCAAAACATTATAATGACAGATGATGGAATCCATCGAAAAATATTCTTACGC |
| tRPS25A | Terminator of putative protein component 25A of the 40S ribosome subunit from *Ogataea polymorpha* (Hanpo2_16366) | ACAGACTAGTATAGTATAATACATGATGCAGAAGTGTTGTAGTACGGTCACAGAGATCTCCAACTCAGGGCGACTTCATCTGGCGTCCTCTTGGACGCGCTTAGCTGCTGACCTGCGGCTGTCCCATTGCCAAATACTACTCTCTCACGGCTTCAAATAATTCACATAAATGCCGATGCGAAAAATTTTACCTCAAAAATATACTGTAAGTACTGATGATGATTGTTCCCCAGAACACTTCGTTGGCGAA |
| tRPS3 | Terminator of putative protein component 3 of the 40S ribosome subunit from *Ogataea polymorpha* (Hanpo2_15182) | GTGTATAGCGCATAATGAACATATAAGTCTATTAGTCTTTAACACCTATGTTGTTCTCACTCATTTGACCAAGCAAACTTTGACCGTTGTCTAAGTTTGGCTCCATATTTATAAAACAAGAGAGGTACAGGAGTTAGGAGAAGTGCAAGGAGCCCTAGCAGCAGGCATGCGTAATGAACTCCTAGTCTCTCGTACATTTGGAGGCCAAAGAGCGGGAAAATTCCAGCCATGGAACATCTTGTAAAGGTGT |
| tPMA1 | Terminator of plasma membrane H+-ATPase from *Ogataea polymorpha* (Hanpo2_88121) | GCCAGCACTTGAATGGGTTAATAGAGGACTGCTCCCCATTATTGATATCTTGCTTCCTTCGTGCCCACGACAGCCTAATAACTAATATTTTGTTCTATTTAGGATGTTCAGTTTTGTGATCTAAGTGTCATTAGCATGATGGATACAATACAATATACCGTTTTTGAATTTTGTAGTATTAATCTGAATATTATTCCTCTGTGGCGGTGCACTCCTGCTTTGTGGCGGTGAAGTCGCGTTTCAGATCAAC |
| tPGK1 | Terminator of putative 3-phosphoglycerate kinase from *Ogataea polymorpha* (Hanpo2_102344) | ACGTGAAATC**TATATA**CTGAG**AAAAAA**AATAATTAATTTTAATATAAACAATGGATCGAGGGGGAAAGAGAATGCCTTTGAGCGAGATTACCGGCAAGCTGGGTCTCAATAGACGGTCGGACCGTGTGTTGAAACCCAACCAGATAAAAATACGGCCTTCCAAGAGCGCCAATCGCGCGATTAACGTGGCAAGTCTCACCAGATCTTATCTCCATAAGGAGATGCAAACACGCACGAAATCCAACATTG |
| tFMD | Terminator of formate dehydrogenase from *Ogataea polymorpha* (Hanpo2_80896) | GCGGTCTTGGAGGAGCTGATTGGATCTAGATGAAATAGGAAATATAATTATGGCTCTACTGCGTCGCGTAAACGTCACTGTAGGCGATTTCGCTTAGCCCCAGTCCGCGATGCGGTCCGACACCAGAGCGCGTCCACCTCCTGTGCGCCGCACCGCCCCCAAAGGAGGTTGCGGCTGTGCGGCTCGACGCGACCAAAAAAATAAGCGTCAAAAGGAGGTGTCAGGGAAGCACGCCGTGGGGCTCGAGATA |
| tCAT | Terminator of peroxisomal catalase from *Ogataea polymorpha* (Hanpo2_95533) | TCATCGATAAGTATTTATTGAATCAGTGTTCGTATACTATATTTTTTTTCATTATCTGCCTGATTTTTTTTTCCCTGATTATAGCTTCCTGATCTGGCAGTCCTGTCCGCCGTATCATCCTTCCTAAAACAGCACGAGATATCGGTATATTATAAGAAGCGTATTTTTCCAATCATTATCTTTATTTTTCATCGTTCATGGCTTGTGGATGTTCCGGTAACAAGAGGACGCAGACCGTCACCGTCCAGCG |
| tTEF1 | Terminator of putative translation elongation factor 1 from *Ogataea polymorpha* (Hanpo2_8811) | ACCACTGCTATTCAAAACAATAGACCATGACTGGGGACTTGGAACAAATATGAAGAGCCACTATTAAATAGCAGTTGAGATCGATTTCACTATCATGCTAAAACTAGCTAGTTCGCATTCAATTCAAATCACTGAATATTTTCATCGAAATGCATGTTGATAAGCCGTCAAAAAATAAATTCGACGACGAATAAACCCATTTGATGAGTTTCCACGAGGTGCTCGACAACGTGACACCTTCAAATTTTCC |
| tTEF2Sc | Terminator of translation elongation factor 2 from *Saccharomyces cerevisiae* | GAGTAATAATTATTGCTTCCATATAATATTTT**TATATA**CCTCTTATTTTTATGTATTAGTTAATTAAGTATTTTTATCTATCTGCTTATCATTTTCTTTTCATATAGGGGGGGTTGGTGTTTTCTTGCCCATCAGATTGATGTCCTCCAACTCGGCACTATTTTACAAAGGGTTTTTTTGTAAGAGAAGGAGAAGACAGATACTAAACCATACGTTACTCGAAAC**AAAAAA**AAAAAAAATGGAAAAAGCTGCTATCAACAAAAGACGGCCTCATC |
| tTEF1Ag | Terminator of translation elongation factor 1 from  *Ashbya gossypii* | ATCAGTACTGAC**AATAAA**AAGATTCTTGTTTTCAAGAACTTGTCATTTGTATAGTTTTTTTATATTGTAGTTGTTCTATTTTAATCAAATGTTAGCGTGATTTATATTTTTTTTCGCCTCGACATCATCTGCCCAGATGCGAAGTTAAGTGCGCAGAAAGTAATATCATGCGTCAATCGTATGTGAATGCTGGTCGCTATACTGCTGTCGATTCGATACTAACGCCGCCATCCAGTGTCGAAAACG |
| tAOXPp | Terminator of alcohol oxidase 1 from *Pichia pastoris* | TCAAGAGGATGTCAGAATGCCATTTGCCTGAGAGATGCAGGCTTCATTTTTGATACTTTTTTATTTGTAACC**TATATA**GTATAGGATTTTTTTTGTCATTTTGTTTCTTCTCGTACGAGCTTGCTCCTGATCAGCCTATCTCGCAGCTGATGAATATCTTGTGGTAGGGGTTTGGGAAAATCATTCGAGTTTGATGTTTTTCTTGGTATTTCCCACTCCTCTTCAGAGTACAGAAGATTAAGTGAGA |

Model for structure of polyA sites in *Saccharomyces cerevisiae*(Guo and Sherman, 1995; Tian and Graber, 2012)

1. **EE: Efficiency element** (25-40 nt upstream of polyA site; can be more distant)

**Consensus: TATATA**

Variations:

TTTTTATA; TAG…TATGTA; TAGTATGTA; TACATA; TATTTA; TATGTT; TATGTA; TTTATA

1. **PE: Positioning element** (10-30 nt upstream of polyA site)
   **Consensus: AATAAA or AAAAAA**Variations: ATTAAGAACG; TTAAGAAC; AAGAA; AATAATGA; TAAATAA

**3) PolyA site
Consensus: Py(A)­_N_**

**Copy number analysis**

To ensure that only a single copy of the respective DNA cassette was integrated in each strain, quantitative real-time PCR (qPCR) was applied. Isolation of genomic DNA for qPCR was performed using the Monarch Genomic DNA Purification Kit (NEB), following the manufacturer’s protocol for yeast cells. Copy number analysis of the integrated cassettes was performed as described by (Abad et al., 2010). For all qPCR reactions the Luna Universal qPCR Master Mix (NEB) was used. As reference genes in *O. polymorpha* TAF10 (Hanpo2_11508) or UBC6 (Hanpo2_99832) were chosen.

To validate the applied qPCR method, the copy number and the correct integration of the cassettes was verified by whole genome sequencing for three exemplary strains (data not shown).

Figure S1: Copy number determination of inserted GFP gene cassettes. For the strains in green the number of GFP cassettes and their correct integration in the genome has been verified by whole-genome sequencing.

*Figure S2: Copy number determination of inserted lacZ-cassette.*For the pCAT_tMOX strain the integration of a single lacZ-cassette at the correct locus was verified by whole-genome sequencing.

**Statistical analysis of promoter and terminator cultivations**

**In the following Tables S5-S8 we list the p-values for each statistical analysis.**

**Table S5.** Unpaired student`s t-test (two-tailed) comparing means for the max RFU of the various promoters and the highest max RFU of the dataset (pMOX on MeOH). A p-value below 0.05 is regarded as significant.

|  | **pMOX** | **pTEF1** | **pTEF2** | **pADH1** | **pDHAS** | **pCAT** |
| --- | --- | --- | --- | --- | --- | --- |
|  |  |  |  |  |  |  |
| **MeOH** |  | 0.017 | 6.0E-07 | 2.2E-06 | 0.003 | 0.207 |
| Significant? |  | **yes** | **yes** | **yes** | **yes** | **no** |
|  |  |  |  |  |  |  |
| **Glucose** |  |  |  |  |  |  |
| 18 hours | 0.0374534 | - | - | - | 0.0000066 | 0.0000003 |
| Significant? | **yes** | **-** | **-** | **-** | **yes** | **yes** |
|  |  |  |  |  |  |  |
| End | 2.08E-06 | 0.00091782 | 0.0009119 | 4.83E-05 | 1.49E-05 | 1.71E-06 |
| Significant? | **yes** | **yes** | **yes** | **yes** | **yes** | **yes** |
|  |  |  |  |  |  |  |
| **Glycerol** | 0.07279 | 0.00084 | 0.00001 | 0.00009 | 0.00001 | 0.00011 |
| Significant? | **no** | **yes** | **yes** | **yes** | **yes** | **yes** |
|  |  |  |  |  |  |  |

**Table S6.** Unpaired student`s t-test (two-tailed) comparing means for the max RFU of the various promoters and the highest max RFU of the dataset (pMOX on MeOH/glycerol). A p-value below 0.05 is regarded as significant.

|  | **pMOX** | **pTEF1** | **pTEF2** | **pADH1** | **pDHAS** | **pCAT** |
| --- | --- | --- | --- | --- | --- | --- |
|  |  |  |  |  |  |  |
| **Glycerol**  **MeOH** |  | 0.00027519 | 0.00015959 | 0.00016264 | 0.00106477 | 0.00195664 |
| Significant? |  | **yes** | **yes** | **yes** | **yes** | **yes** |
|  |  |  |  |  |  |  |
| **Glucose**  **MeOH** | 5.7E-05 | 0.00028150 | 4.9E-06 | 0.00022436 | 6.8E-05 | 0.00013008 |
| Significant? | **yes** | **yes** | **yes** | **yes** | **yes** | **yes** |

**Table S7 -** Unpaired student`s t-test (two-tailed) comparing means for the max RFU of the various terminator strains to the highest max RFU of the dataset (tMOX) on each carbon source. A p-value below 0.05 is regarded as significant.

|  | **tTEF2_Sc** | **tAOX_Pp** | **tAMO** | **tTDH3** | **tRPS2A** | **tPMA1** |
| --- | --- | --- | --- | --- | --- | --- |
|  |  |  |  |  |  |  |
| **MeOH** | 5,92E-08 | 1,00E-05 | 7,53E-08 | 4,00E-07 | 9,67E-08 | 3,68E-07 |
| Significant? | **yes** | **yes** | **yes** | **yes** | **yes** | **yes** |
|  |  |  |  |  |  |  |
| **Glucose** | 2,21E-05 | 3,28E-06 | 1,01E-06 | 3,77E-06 | 1,01E-07 | 9,26E-07 |
| Significant? | **yes** | **yes** | **yes** | **yes** | **yes** | **yes** |
|  |  |  |  |  |  |  |
| **Glycerol** | 2,67E-06 | 6,10E-05 | 1,08E-06 | 1,99E-06 | 1,56E-06 | 1,01E-06 |
| Significant? | **yes** | **yes** | **yes** | **yes** | **yes** | **yes** |
|  |  |  |  |  |  |  |
|  |  |  |  |  |  |  |
|  | ***tFMD*** | ***tCAT*** | ***tTEF1*** | ***tTEF1_Ag*** | ***tCYC1*** | ***tRPS25A*** |
|  |  |  |  |  |  |  |
| **MeOH** | 1,96E-06 | 2,42E-07 | 1,04E-05 | 5,14E-08 | 6,08E-08 | 1,28E-07 |
| Significant? | **yes** | **yes** | **yes** | **yes** | **yes** | **yes** |
|  |  |  |  |  |  |  |
| **Glucose** | 8,19E-06 | 7,74E-08 | 7,60E-08 | 2,51E-07 | 3,58E-08 | 5,91E-07 |
| Significant? | **yes** | **yes** | **yes** | **yes** | **yes** | **yes** |
|  |  |  |  |  |  |  |
| **Glycerol** | 1,17E-06 | 1,06E-06 | 1,40E-06 | 1,14E-06 | 5,21E-07 | 1,31E-06 |
| Significant? | **yes** | **yes** | **yes** | **yes** | **yes** | **yes** |
|  |  |  |  |  |  |  |
|  |  |  |  |  |  |  |
|  | ***tRPS3*** | ***tPGK1*** |  |  |  |  |
|  |  |  |  |  |  |  |
| **MeOH** | 7,54E-08 | 4,69E-07 |  |  |  |  |
| Significant? | **yes** | **yes** |  |  |  |  |
|  |  |  |  |  |  |  |
| **Glucose** | 1,10E-07 | 4,69E-07 |  |  |  |  |
| Significant? | **yes** | **yes** |  |  |  |  |
|  |  |  |  |  |  |  |
| **Glycerol** | 1,39E-06 | 6,86E-07 |  |  |  |  |
| Significant? | **yes** | **yes** |  |  |  |  |

**Table S8.** Unpaired student`s t-test (two-tailed) comparing means for the max RFU of the various promoters-terminator pairs and the highest max RFU of the dataset (pMOX or pCAT). A p-value below 0.05 is regarded as significant.

|  | **pMOXtMOX** | **pMOXtCAT** | **pMOXtTEF1** | **pCATtMOX** | **pCATtCAT** | **pCATtTEF** |
| --- | --- | --- | --- | --- | --- | --- |
|  |  |  |  |  |  |  |
| **GFP MeOH** |  | 2.8682E-05 | 0.00022619 |  | 4.17803E-07 | 2.80659E-08 |
| Significant? |  | **yes** | **yes** |  | **yes** | **yes** |
|  |  |  |  |  |  |  |
| **GFP Glycerol** |  | 0.00021546 | 4.93E-06 |  | 0,00014713 | 8,60E-05 |
| Significant? |  | **yes** | **yes** |  | **yes** | **yes** |
|  |  |  |  |  |  |  |
| **lacZ**  **MeOH** |  | 0.0001465 | 0.00010958 |  | 0.00294125 | 0.00339650 |
| Significant? |  | **yes** | **yes** |  | **yes** | **yes** |

**Growth of *O. polymorpha***





*Figure S3. Growth rates of* O. polymorpha *on different carbon sources.* O. polymorpha *was cultivated on Verduyn minimal medium containing 15 g/L glucose, glycerol or methanol in a cell growth quantifier (CGQ) devices which measures the scattered light value over the time course of the batch cultivation. Growth rates (µ) were calculated from the exponential growth phase for each carbon source.*

**Substrate Consumption**





*Figure S4 – Substrate consumption of* O. polymorpha *on different carbon sources.* O. polymorpha *was cultivated in the BioLector on Verduyn minimal medium containing 15 g/L glycerol or 15 g/L methanol. Substrate concentrations were quantified using HPLC for the original cultivation medium (0 h, 1 replicate) and after the cultivation (150 h, 3 biological replicates). In all tested cultures the carbon source was consumed entirely.*


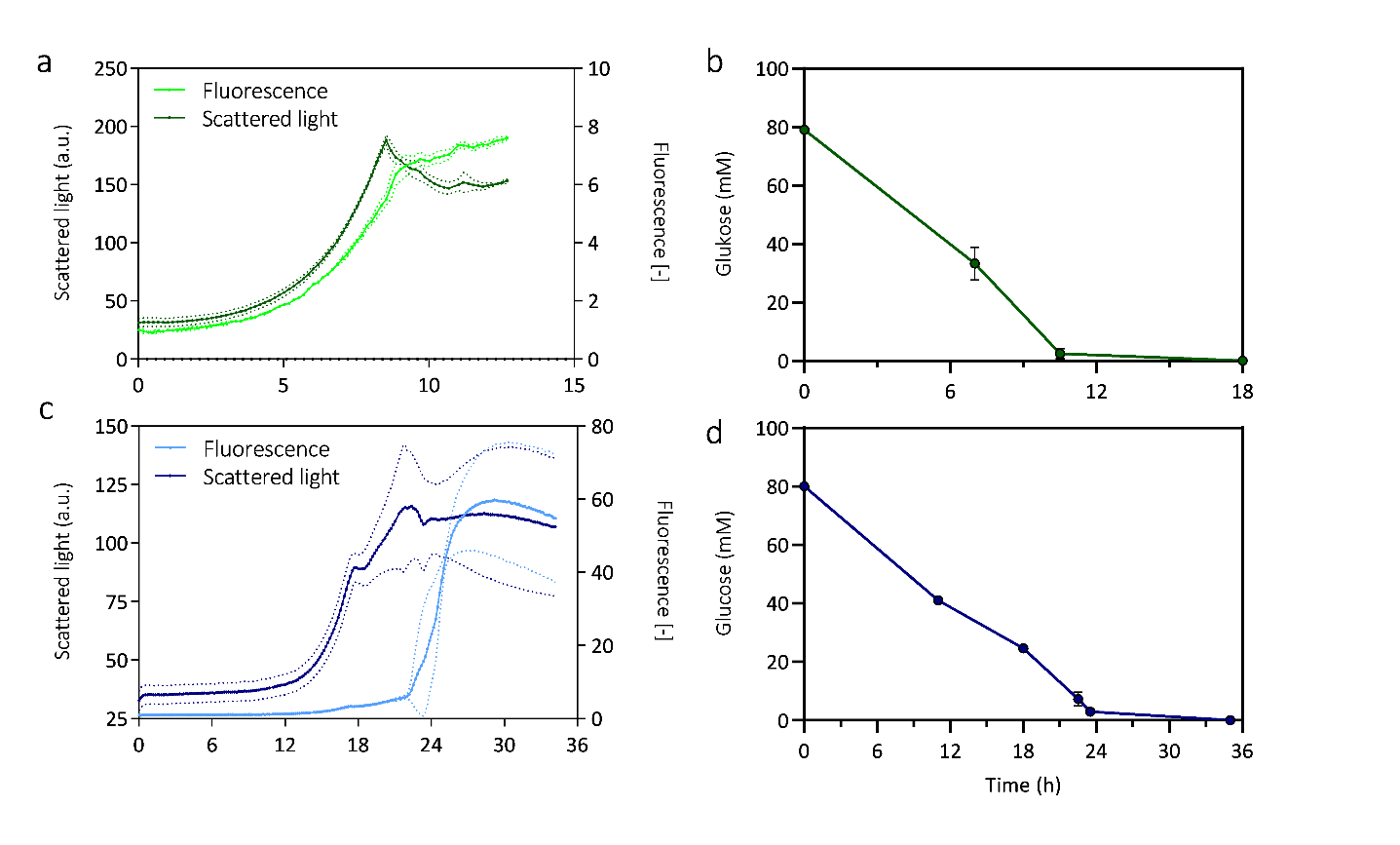


*Figure S5 – Substrate consumption of* O. polymorpha *on glucose.* O. polymorpha *was cultivated in the BioLector on Verduyn minimal medium containing 15 g/L glucose. Growth (Scattered light) and fluorescence (****a,c****) for* O. polymorpha*_NCYC495_yku80_leu- (****a****) and* O. polymorpha*_NCYC495_yku80_leu-_pMOX-ubiGFP_tAMO (****c****) were measured in the BioLector device. Glucose consumption (****b,d****) of* O. polymorpha*_NCYC495_yku80_leu- (****b****) and* O. polymorpha*_NCYC495_yku80_leu-_pMOX-ubiGFP_tAMO (****d****) was measured via HPLC.*

**Statistical evaluation of mRNA analyses**

**Paired t-test (two-tailed) comparing means of the transcript abundance in the tMOX and tCYC1 strains**

Testing assumptions made in a paired two-tailed t-test.

1. The differences are distributed according to a Gaussian distribution.

To test this assumption for the data set a Shapiro-Wilk normality test was performed

|  | **tMOX** | **tCYC1** |
| --- | --- | --- |
| **P value** | 0,1839 (ns) | 0,2615 (ns) |
| **Passed normality test? (alpha=0.05)** | Yes | Yes |

Thus, the sample data does not deviate significantly from a Gaussian distribution.

1. Test for effectiveness of pairing with Pearson correlation coefficient

|  | **tMOX vs tCYC1** |
| --- | --- |
| **Correlation coefficient (r)** | 0,8785 |
| **P value** | 0,0041 |

The two groups are significantly correlated, the use of a paired t-test is justified.

1. **Paired t-test (two-tailed):**

| **Timepoint [min]** | **tMOX** | **tCYC1** |
| --- | --- | --- |
|  | **Mean** | **Mean** |
| **0** | 431,56 | 143,40 |
| **2** | 403,21 | 177,53 |
| **5** | 488,9 | 167,84 |
| **10** | 576,58 | 262,03 |
| **15** | 917,46 | 348,43 |
| **30** | 631,61 | 359,19 |
| **45** | 971,43 | 547,06 |
| **60** | 887,89 | 632,12 |
| **P value: < 0.0001** | | |

The means of the two groups differ significantly.

**Comparing the means of the transcript abundance in the tMOX and tCYC1 strain for each measuring time point separately with a paired two-tailed t-Test.**

| **Timepoint [min]** | **tMOX vs. tCYC1** |
| --- | --- |
|  | **p-value** |
| 0 | <0.0001 |
| 2 | 0.0005 |
| 5 | 0.0002 |
| 10 | 0.0002 |
| 15 | 0.0140 |
| 30 | 0.001 |
| 45 | 0.0432 |
| 60 | 0,0705 |

**Paired t-test (two-tailed) comparing means for tMOX and tCYC1 relative transcript decay**

Testing assumptions made in a paired t-test.

1. The differences are distributed according to a Gaussian distribution.

To test this assumption for the data set a Shapiro-Wilk normality test was performed

|  | **tMOX** | **tCYC1** |
| --- | --- | --- |
| **P value** | 0,437 (ns) | 0,334 (ns) |
| **Passed normality test? (alpha=0.05)** | Yes | Yes |

Thus, the sample data does not deviate significantly from a Gaussian distribution.

1. Test for effectiveness of pairing with Pearson correlation coefficient

|  | **tMOX vs tCYC1** |
| --- | --- |
| **Correlation coefficient (r)** | 0.901 |
| **P value** | 0.003 (*) |

The two groups are significantly correlated, the use of a paired t-test is justified.

**Paired t-test (two-tailed):**

| **Timepoint [min]** | **tMOX** | **tCYC1** |
| --- | --- | --- |
|  | **Mean** | **Mean** |
| **2** | 1.007 | 0.796 |
| **5** | 0.841 | 0.889 |
| **10** | 0.717 | 0.543 |
| **15** | 0.420 | 0.382 |
| **30** | 0.655 | 0.382 |
| **45** | 0.392 | 0.250 |
| **60** | 0.444 | 0.210 |
| **P value: < 0.015** | | |

The means of the two groups differ significantly.

**Comparing the means of the relative transcript decay in the tMOX and tCYC1 strain for each measuring time point separately with a paired two-tailed t-Test.**

| **Timepoint [min]** | **tMOX vs. tCYC1** |
| --- | --- |
|  | **p-value** |
| 2 | 0,114 |
| 5 | 0,569 |
| 10 | 0,030 |
| 15 | 0,602 |
| 30 | 0,004 |
| 45 | 0,104 |
| 60 | 0,010 |

**Paired t-test (two-tailed) comparing max RFU of the various promoters-terminator pairs and the highest max RFU of the dataset**

**References**

Abad, S., Kitz, K., Hörmann, A., Schreiner, U., Hartner, F. S., and Glieder, A. (2010). Real-time PCR-based determination of gene copy numbers in *Pichia pastoris*. *Biotechnol. J.* 5, 413–420. doi:10.1002/biot.200900233.

Apel, A. R., Espaux, L., Wehrs, M., Sachs, D., Li, A., Tong, G. J., et al. (2017). A Cas9-based toolkit to program gene expression in *Saccharomyces cerevisiae*. 45, 496–508. doi:10.1093/nar/gkw1023.

Guo, Z., and Sherman, F. (1995). 3’-end-forming signals of yeast mRNA. *Mol. Cell. Biol.* 15, 5983–5990. doi:10.1128/mcb.15.11.5983.

Saraya, R., Krikken, A. M., Veenhuis, M., and van der Klei, I. J. (2011). Peroxisome reintroduction in *Hansenula polymorpha* requires Pex25 and Rho1. *J. Cell Biol.* 193, 885–900. doi:10.1083/jcb.201012083.

Tian, B., and Graber, J. H. (2012). Signals for pre-mRNA cleavage and polyadenylation. *Wiley Interdiscip. Rev. RNA* 3, 385–396. doi:10.1002/wrna.116.
